# Early intervention with Kan Jang® to treat upper-respiratory tract infections: A randomized, quadruple-blind study

**DOI:** 10.1016/j.jtcme.2021.06.001

**Published:** 2021-06-11

**Authors:** Mikayel Narimanyan, Kristina Jamalyan, Anna Balyan, Anders Barth, Staffan Palm, Georg Wikman, Alexander Panossian

**Affiliations:** a Yerevan State Medical University of Armenia, Koryun 2, RA-0025 Yerevan, Armenia; b Erebouni Medical Center, 14 Titogradyan Str, RA-0087 Yerevan, Armenia; c Partus Kvinnohälsa, Korsgatan 4, SE-411 16 Göteborg, Sweden; d Panafarma Medical Consult AB, Skottorps Castle, SE-312 96 Laholm, Sweden; e SHI Research and Development AB, Kövlingevägen 21, SE-312 50 Vallberga, Sweden; f Phytomed AB, Bofinkvägen 1, Våxtorp 31275, Sweden

**Keywords:** Kan Jang®, *Andrographis paniculata*, *Eleutherococcus senticosus*, Clinical trial, Upper-respiratory tract infections, Inflammation., ESR, erythrocyte sedimentation rate, FI, farb (colour) index, GCP, good clinical practice, GMP, good manufacturing practice, ICH, international conference on harmonization, OR, odds ratio, QP, qualified pharmacist, RBC, red blood cell, TSS, total symptom score, URTI, upper respiratory tract infection, VAS, visual analog scale, WBC, white blood cell

## Abstract

**Background and aim:**

*Andrographis paniculata* and *Eleutherococcus senticosus* preparations and their fixed combination, called Kan Jang®, are traditionally used for relieving symptoms of upper-respiratory tract infections (URTIs). This study aimed to assess the efficacy of early intervention with Kan Jang® on the relief and duration of inflammatory symptoms during the acute phase of the disease.

**Experimental procedure:**

A total of 179 patients with URTI symptoms received six Kan Jang® (daily dose of andrographolides: 60 mg) or placebo capsules a day for five consecutive days in this randomized, quadruple-blinded, placebo-controlled, two-parallel-group phase II study. The primary efficacy outcomes were the decrease in the acute-phase duration and the mean URTI symptoms score (sore throat, runny nose, nasal congestion, hoarseness, cough, headache, and fatigue).

**Results:**

Early intervention with Kan Jang® significantly increased the recovery rate and reduced the number of sick leave days by >21% (0.64/day) relative to that observed in the placebo group (2.38 vs. 3.02 days, p = 0.0053). Kan Jang® significantly alleviated all URTI symptoms starting from the second day of treatment. A superior anti-inflammatory effect of Kan Jang® to that of placebo was also observed on the white blood cell count (p = 0.007) and erythrocyte sedimentation rate (p = 0.0258). Treatment with Kan Jang® was tolerated well.

**Conclusion:**

This study demonstrates that early intervention with Kan Jang® capsules reduces the recovery duration of patients by 21% and significantly relieves the severity of typical URTI symptoms.

## Highlights

Early intervention with Kan Jang® capsules, a fixed combination of *Andrographis paniculata and Eleutherococcus senticosus extracts:*• Significantly increased patients' recovery rate on days 2, 3, and 4 compared to placebo.
• Increased the number of recovered patients by 24% on day 3 compared to placebo control.
• Reduced the time to major improvement, defined as the number of sick leave days at home.
• Reduced the overall recovery duration time of patients by 21%.
• Significantly relieved typical URTI symptoms severity.

## Graphical abstract

**Figure:**
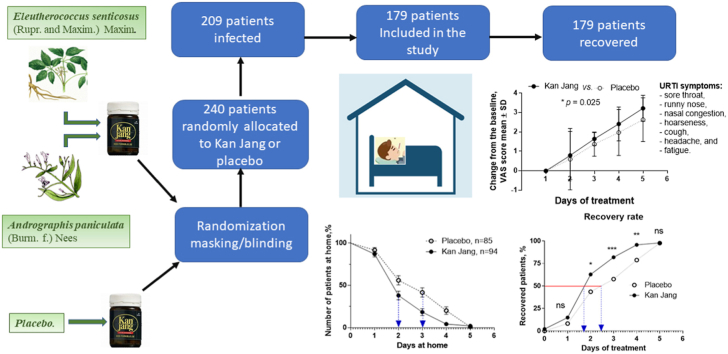

## 1 Introduction

Upper-respiratory tract infectious (URTI) diseases, such as rhinitis, sinusitis, nasopharyngitis (common cold), and laryngitis, are some of the most common diseases and are characterized by an acute self-limited defense response onset between 1 and 3 days after viral exposure and inflammatory symptoms, such as cough, sneezing, nasal discharge, nasal congestion, runny nose, sore throat, and nasal breathing lasting 1–2 weeks.^1^^,^^2^ Although most URTIs are mild, they are the main reason for absence from school or work.^2^ Antibiotics are usually helpful in treating bacterial infections predominantly of the lower respiratory tract airways but not against viral URTI.^3^^,^^4^ Increasing evidence suggests that some herbal preparations are effective and well-tolerated medications for preventing and treating acute viral respiratory infections.^5^^,^^6^ Thus, the herbal medicinal product Kan Jang®, a fixed combination of a proprietary blend of extracts of *Andrographis paniculata* L. (Burm. F.) Wall. ex. Nees (SHA-10) and *Eleutherococcus senticosus* (Rupr. & Maxim.) Maxim (SHE-3) has been used in Scandinavia for treating symptoms of viral respiratory diseases, such as common colds and influenza, for >30 years.^1^^,^^7^, ^8^, ^9^, ^10^, ^11^

In a multicenter pilot study conducted in Sweden (unpublished in-home report from 2009), a significant reduction in sick leave days at home was observed in the Kan Jang® group compared with those in the placebo group. Based on the results of that unpublished study, we, for the first time, hypothesize that (i) Kan Jang decreases sick days at home, increasing the rate of recovery and that (ii) Kan Lang is significantly effective compared with placebo during the early stage of the disease.

The aim of this randomized, quadruple-blinded, placebo-controlled, two-parallel-group phase II study was to assess the efficacy of Kan Jang®, with the duration of sick leave at home as the objective primary outcome measure reflecting the rate of recovery. Moreover, relief of URTI symptoms severity, particularly during the early stages of URTIs, was used as a secondary efficacy outcome measure.

## 2 Materials and methods

### 2.1 Study design, recruitment, and screening of patients, schedule of examinations

**Fig. 1:**
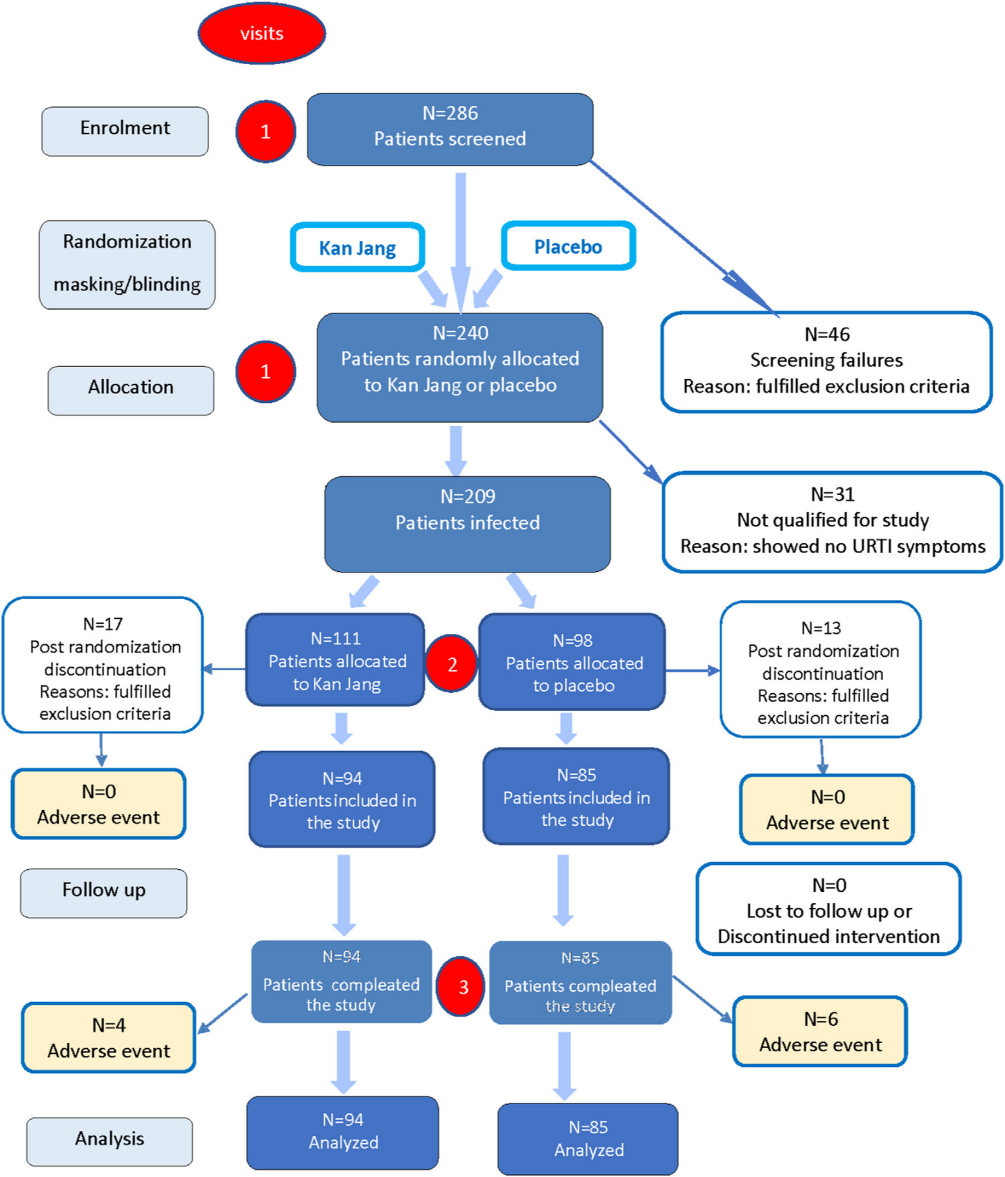
Schematic diagram of the trial. CONSORT flow chart of the disposition of patients in the two study arms. The figure shows the numbers of randomized patients that entered, discontinued, and completed the study as well as the adverse events and reasons for all postrandomization discontinuations.

This prospective, randomized, placebo-controlled, quadruple-blind, two-parallel-group (Fig. 1 and Supplement 1), phase II interventional study was conducted at the Family Doctor Department of Yerevan State Medical University from January 2011 to December 2012. The study was approved by the Ethics Committee Board and the authorities (Registration Nr 10, date of approval of final protocol 2012-01-03). They reviewed the Study Protocol and the Investigator's Brochure. The trial was conducted according to Good Clinical Practices (GCP) and GCP International Conference on Harmonization (ICH) Guidelines, FDA July 17, 1996 (61 FR 37319).

Information about the study was provided to the study participants in Armenian and English languages, as per local regulations.

The schedule of examinations and procedure evaluations included three visits (Table 1): a medical examination and assessment for eligibility (visit #1), the final inclusion and evaluation of primary and secondary baseline points (visit #2), and evaluation of primary and secondary endpoints (visit #3). During visit #1, the patients were informed about the study details and provided study medication and self-assessment questionnaires. Each patient was randomly assigned to the study medication. All subjects started taking the study preparation as soon as the first symptoms of URTI appeared and immediately contacted the doctor for visit #2, during which the doctors assessed them for inclusion in the study (Fig. 1). The doctors recorded the baseline primary and secondary outcome measures and took blood samples. After visit #2, each patient was instructed to fill in the self-assessment questionnaire for the next 4 days. During the last visit (visit #3), after 5 days of treatment, the final medical examination and the second sampling of blood were performed. The final primary and secondary endpoints were recorded. The number of consumed capsules during treatment was verified for every patient for compliance. The duration of the treatment and illness were recorded as days at home.

**Table 1:** Schedule of assessment.

Assessment	Screening	Treatment				
		Baseline	Days			
**Study day**	**0**	**1**	**2**	**3**	**4**	**5**
**Visit Nr**	**1**	**2**				**3**
Informed consent	∗					
History	∗					
Physical examination	∗	∗				
Primary outcome measure		∗	∗	∗	∗	∗
Secondary outcome measures		∗	∗	∗	∗	∗
Laboratory tests		∗				∗
Adverse events		∗	∗	∗	∗	∗

### 2.2 Selection of the study population

#### 2.2.1 Inclusion and exclusion criteria

The target population was patients (males and females) aged 18–65 years with symptoms of uncomplicated acute upper-respiratory infections (URI; J00–J04 according to the ICD-10, 10th Revision; ICD-10 Version: 2010 (who.int)), including acute nasopharyngitis (common cold), sinusitis, pharyngitis, tonsillitis, laryngitis, and tracheitis. Patients affected by the first signs and symptoms of URTI, such as sore throat, blocked nose, runny nose, hoarseness, cough, headache, and general malaise, were included in the study after visit #2.

Exclusion criteria at visit #1: any known abuse of medication, narcotics, tobacco (>20 cigarettes/day), or alcohol; known allergies against cut flowers, herbal spices, or bitter seeds; pregnant or breast-feeding or trying to get pregnant; or regularly taking any kind of medication that can affect the course of an infection (i.e., anti-inflammatory medicines, antihistamines, or corticosteroids). There were 46 participant dropouts at the beginning of the study (visit #1**,** Fig. 1**,** pre-randomization discontinuation).

Exclusion criteria at visit # 2: symptoms of a common cold for >36 h, fever >38.5 °C, accompanying infections requiring treatment with antibiotics, taking any medication that can affect the course of the disease (i.e., anti-inflammatory medicines, antihistamines, corticosteroids, or antibiotics). During visit #2, 30 out of 209 infected patients were excluded from the study (Fig. 1, post-randomization discontinuation) because 31 recruited volunteers showed no URTI symptoms.

The participants were free to withdraw from the study at any time without giving a reason or due to serious side effects, an allergy or hypersensitivity to study medication, or britches exclusion criteria during the study.

### 2.3 Study medication

#### 2.3.1 Intervention and comparators

Pharmaceutical-grade standardized extracts of *Andrographis paniculata* L. Nees. (herb) and *Eleutherococcus senticosus* (Rupr. & Maxim.) Maxim (root) genuine extracts as well as their fixed combination, Kan Jang®/agaDA®, were manufactured, tested, and then released for human use as per ICH Q7A and EMEA guidelines for Good Agricultural and Collecting Practice and Good Manufacturing Practice (GMP) of active pharmaceutical ingredients at the Swedish Herbal Institute, which holds a valid EU-GMP license to produce pharmaceuticals.

One capsule of Kan Jang® (size 1, batch No. 50145) contains 195 mg of *A. paniculata* herbal native extract SHA-10 (batch no. 1521114, drug-native extract ratio of 6.7: 1, extraction solvent 70% ethanol), standardized to 10 mg of diterpene lactones andrographolide, 14-deoxy-11,12-didehydroandrograholide, and 11.4 mg of *E. senticosus* root quantified native extract (batch no. 1521077, drug-native extract ratio of 17: 1, extraction solvent 70% ethanol). The details of the batch analysis are shown in Supplement 2**.** The matrix contained inactive excipients (microcrystalline cellulose and magnesium stearate). The placebo capsules containing the inactive excipients were identical to the Kan Jang® capsules. The appearance, smell, and color of both preparations were similar and organoleptically undistinguishable.

#### 2.3.2 Study medication quality assurance

Herbal preparations were qualitatively and quantitatively tested via thin-layer chromatography and high-performance liquid chromatography as per specifications using appropriate reference standards (Supplement 2). The analytical methods were validated for selectivity, accuracy, and precision. The intervention and comparators were packed and labeled by the Swedish Herbal Institute as per national requirements regarding their use in clinical trial investigations. Moreover, the label contained the drug name, study code, and storage conditions. Reference samples were retained and kept at the QC Laboratory of the Swedish Herbal Institute.

#### 2.3.3 Doses, treatment regimens, evaluation of compliance

The daily dose of the study intervention was two capsules thrice per day for five consecutive days. This treatment dose corresponded to the dose used in earlier studies, with a daily intake of 1170 mg of the dry extract *A. paniculata* SHA-10, corresponding to 60 mg andrographolides and 68.4 mg of dry extract of *E. senticosus*, in the Kan Jang® group. All the patients were provided with diary cards on which the daily consumption of study medication was recorded. The number of consumed medications during treatment by every single patient was verified for compliance with the duration of the treatment/illness (days at home). The investigator was responsible for maintaining drug accountability records.

#### 2.3.4 Randomization, allocation concealment, and blinding

A randomization sequence table containing two columns (A and B) filled with randomly distributed unique numbers from 1 to 240 was generated by a qualified pharmacist (QP) using an Excel random number generator at the manufacturing site before the study.

It contains information about the distribution of unique numbers between groups A and B.

The treatment code disclosing the actual assignment of Kan Jang and placebo capsules to A and B groups/sets of packages was encoded by a QP during the study medication (treatment) randomization procedure at the manufacturing site.

Study preparations (placebo and Kan Jang® capsules) were labeled by a QP at the manufacturing site using a treatment randomization sequence and treatment code.

The principal investigator generated a study participants list that identified all the patients randomly assigned to treatments encoded by random treatment code numbers. The principal investigator wrote the patient's name on the package labels and in the case report forms, where the corresponding treatment code was recorded. Thus, the study participants list, identifying the patients and the study medication packages (treatment code numbers), was provided for statistical analyses at the end of the study.

The treatment randomization sequence was kept confidential by a QP at the manufacturing site and was provided to the principal investigator for statistical evaluation of the results when all patients completed the treatment.

A QP disclosed the treatment code, providing the information about the actual assignment of groups A and B to Kan Jang and placebo after statistical analysis of the results.

Overall, the masking of organoleptically identical Kan Jang and placebo capsules was quadruple blind in participants, care providers, investigators, and outcomes assessors.

### **2.4** Efficacy and safety endpoints

The efficacy and safety of the treatment were evaluated using the results of the medical observations and the patient-reported self-assessment questionnaires.

#### 2.4.1 Efficacy primary outcomes

The sick leave days at home were an absolute parameter that gave a clear indication of the patient's general state of disease and ability to perform. Thus, the primary outcome measure was the incidence of sick leave at home in the intervention group and was compared with that in the control group during the first 3–5 days of treatment. The second primary outcome measure was the duration of sick leave at home (the acute phase of the disease) in the intervention group and was compared with that in the control group.

The study's primary endpoint was the difference in the proportion of participants with a poor outcome, defined as the duration of sick leave at home during the first 3–5 days between the Kan Jang® and placebo groups. The patients were instructed to record each day of absence from work.

#### 2.4.2 Efficacy secondary endpoints

The secondary efficacy endpoints of the study were the differences in the relief of inflammatory symptoms (sore throat, runny nose, nasal congestion, hoarseness, cough, headache, and fatigue) in the Kan Jang® and placebo groups. The secondary outcomes were changes in the severity of inflammatory symptoms measured using the Visual Analog Scale (VAS) scores from the baseline to the end of therapy (day 5). These outcomes were calculated as the mean of all symptoms scores, the Total Symptom Score (TSS) for days 2–4 of the 5-day treatment period. The TSS is the sum of the seven individual symptom scores (sore throat, nasal obstruction, nasal discharge, cough, hoarseness, headache, and general malaise). The symptoms were evaluated daily using a 10-point score scale, with the score of 0 = no URTI symptoms, scores 1–3 = mild symptoms, scores 4–6 = moderate symptoms, and scores 7–9 = severe symptoms.

Surrogate outcome variables were a general blood analysis, including hemoglobin (Hb) in g/L, red blood cells in 10^12^/L, farb (colour) index (FI), platelets in 10^9^/L, white blood cells (WBCs) in 10^9^/L, stab neutrophils in percent, segmented neutrophils in percent, eosinophils in percent, lymphocytes in percent, monocytes in percent, and erythrocyte sedimentation rate (ESR) in mm/h.

#### 2.4.3 Safety outcomes

Safety and tolerability were assessed by monitoring the frequency, duration, and severity of adverse events (Supplement 4).

### 2.5 Statistical analysis

Statistical analysis was performed using GraphPad Prism Software 3.0 (GraphPad Software Inc., San Diego, CA, USA). The results and details are shown in Supplement 3. Statistical assessment of the baseline characteristics and comparison of the two parallel groups were performed using column statistics, including the D'Agostino & Pearson omnibus normality test. Based on the normality test results, either an unpaired *t*-test (for variables with a normal distribution) or the Mann–Whitney nonparametric test was applied (Table 2 and Table 1 in Supplement 3).

**Table 2:** Baseline demographic and efficacy outcome measures, mean ± SE.

	Placebo, n = 85	Kan Jang®, n = 94	Mean difference	p-value
Age, years	34.08 ± 1.52	35.62 ± 1.314	−0.164	0.3814
Males/Females	41/44	47/47	6/3	>0.05
Sore throat	5.471 ± 0.057	5.415 ± 0.055	0.056	0.4030
Runny nose	5.647 ± 0.052	5.564 ± 0.054	0.083	0.3075
Nasal congestion	5.659 ± 0.052	5.553 ± 0.051	0.106	0.2148
Hoarseness	5.612 ± 0.056	5.479 ± 0.058	0.133	0.1626
Cough	5.600 ± 0.058	5.489 ± 0.056	0.111	0.2503
Headache	5.365 ± 0.076	5.340 ± 0.056	0.025	0.5938
Fatigue	5.388 ± 0.075	5.351 ± 0.056	0.037	0.5200
**Total mean**	**5.535 ± 0.047**	**5.456 ± 0.090**	0.079	0.2086

Abbreviations: SE, standard error of the mean. Baseline blood hematology analysis results at the beginning of URTI are shown in Supplement 5.

**Fig. 2:**
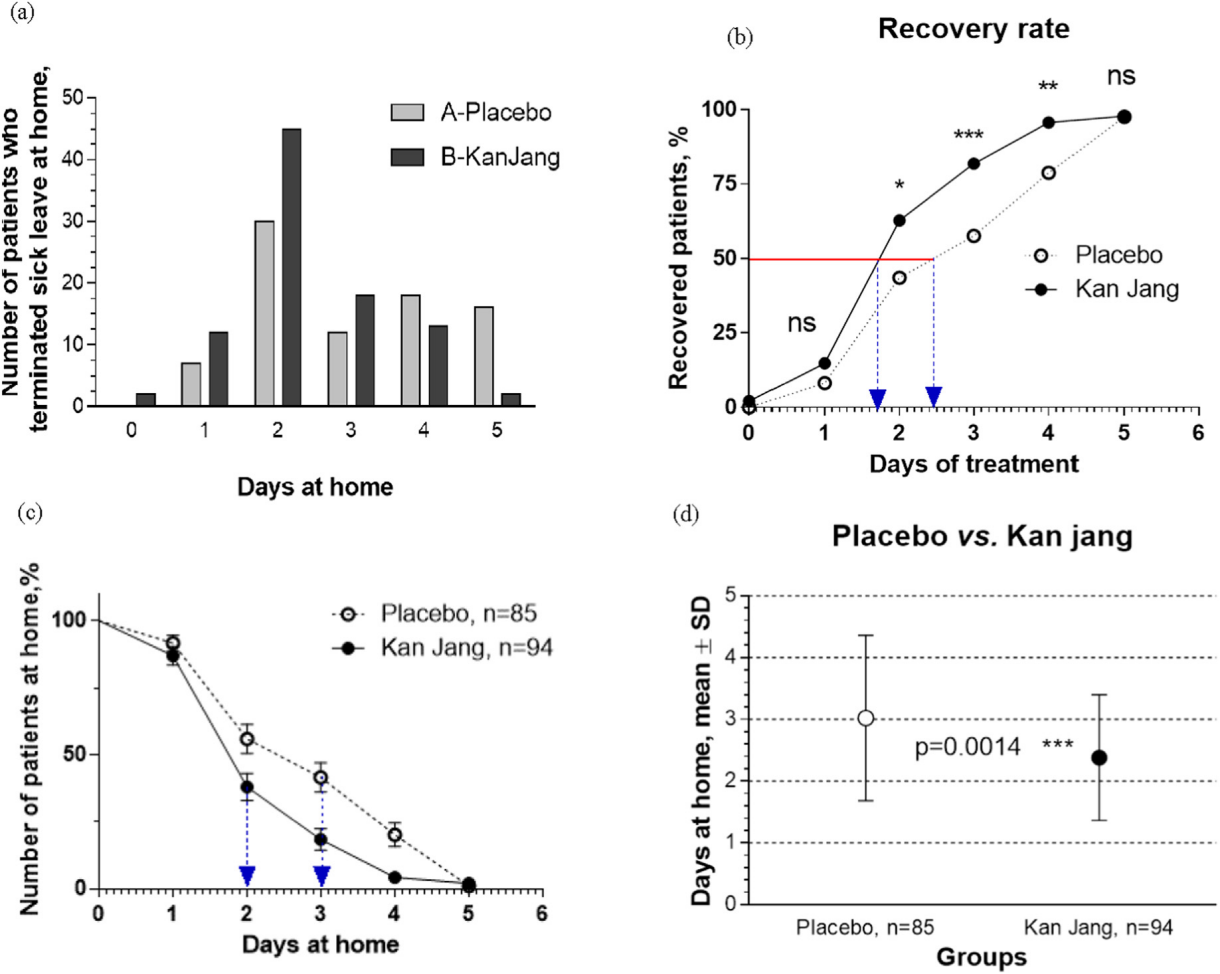
Primary efficacy outcome: (a) Number of patients who terminated sick leave at home in the Kan Jang® and placebo groups. (b) The time-dependent plot of the cumulative number of patients in the Kan Jang® and placebo groups who terminated sick leave at home during the trial, with a statistically significant difference in the recovery rates (recovered patients [%]/time of treatment) of patients observed on days 2, 3, and 4 between the groups (∗p < 0.05, ∗∗p < 0.01, ∗∗∗p < 0.001, OR and z-statistics, Supplement 3). Sick leave at home was terminated by 50% of the patients in 1.7 days in the Kan Jang® group and 2.5 days in the placebo group. (c) Kaplan–Meier estimates the incidences of sick leave at home in Kan Jang® group B and placebo group A during the first 5 days of treatment. The number of days at home was three in A and two in B; median A/B ratio, 1.5; hazard A/B ratio, 0.495; and (d) the mean duration of sick leave at home was 2.3 days in the Kan Jang® group and 3.0 days in the placebo group, p = 0.0014.

The same approach (column statistics, normality test followed by the Mann–Whitney or unpaired *t*-test) was applied for assessment of statistically significant differences between the groups at the end of the treatment performed by comparing the means of the duration of sick leave at home (Fig. 2d) and of the blood analysis tests results (Table 3).

**Table 3:** Changes from the baseline blood hematology analysis result on the day 5 endpoint.

	Placebo, n = 85		Kan Jang®, n = 94		p-value
	Mean	SE	Mean	SE	
FI	−1.753e−01	0.003	−0.002	0.005	0.992
WBCs 10^9^/L	−0.549	0.108	−0.706	0.120	**0.007∗∗**
Stab neutrophils %	−0.659	0.113	−0.723	0.112	0.604
Segmented neutrophils %	−0.235	−0.235	0.681	0.591	0.637
Eosinophils %	−0.435	0.068	−0.425	0.063	0.782
Lymphocytes %	−0.047	0.202	0.702	0.261	0.149
Monocytes %	−0.212	0.053	−0.298	0.052	0.407
ESR mm/h	−2.118	0.223	−2.819	0.208	**0.026∗**

WBC, white blood cells; STN and SGN, stab and segmented neutrophils; EOS, eosinophils; L, lymphocytes; M, monocytes; ESR, erythrocytes sedimentation rate; FI, colored index; SE, standard error of the mean.

The primary endpoint was defined as the duration of sick leave at home. Kaplan–Meier curves were generated for all endpoints, and medians were calculated from those curves. The treatment arms were compared by performing the Mantel-Cox log-rank and Gehan–Breslow-Wilcoxon tests. The estimates of treatment hazard ratios based on the log-rank tests and 95% CIs were calculated (Fig. 2c and Supplement 3).

Intergroup comparison of the number of patients who took sick leave at home was assessed using the odds ratio (OR) statistics of endpoints according to the Bland–Altman A/B test of significance of differences of endpoints at 95% confidence and the z-statistics at 95% confidence of statistical significance (Fig. 2b and Supplement 3).

The treatment efficacies over time were measured using the change from the baseline (visit 2) to each scheduled measurement during the study, including the last visit to the doctor (visit 3; endpoint). The statistical significance of differences between the effects of Kan Jang® and placebo over time were assessed using two-way between–within ANOVA wherein an interaction effect indicates a different response over time between the two groups, and thus would signal a treatment effect (Fig. 4) as well as by a multiple-comparison *t*-test (one unpaired test per row) (see Supplement 3).

The incidences of adverse events were compared across treatment groups for descriptive purposes and to identify possible differences in the safety profiles by using OR statistics of endpoints according to the Altman A/B test of significance of differences of endpoints at 95% confidence and z-statistical methods for categorical data https://www.medcalc.org/calc/odds_ratio.php.

#### 2.5.1 Sample size considerations

The sample size was determined according to the effect size from a comparable study^10^ of Kan Jang® in URTIs wherein inflammatory symptoms were used as efficacy outcome measures. A minimum of 120 participants was required to detect a significant difference between the Kan Jang® and placebo groups with a 95% power and at an α-error probability 0.05 level of significance (Stat-Mate, version 2.00, 2004; GraphPad Software, Inc.). We prepared for a significant dropout rate by increasing our intended sample size to 240 participants.

## 3 Results

### 3.1 Baseline demographic and clinical characteristics

As the main objective of the study was to evaluate the effectiveness of early intervention with Kan Jang® on the relief of URTI symptoms, 286 potential patients were screened for compliance with the inclusion criteria, and 240 healthy subjects were recruited (visit #1) in the study before the onset of URTI symptoms (Fig. 1). Post randomization inclusion was performed on the first day of URTI symptoms (visit #2), and 30 patients were excluded from the study because they did not meet the inclusion criteria (experiencing common cold symptoms for >36 h, body temperature >38.5 °C, requiring antibiotic treatment or taking other medications that can affect the course of infection). Moreover, 31 of the 240 recruited subjects had no common cold symptoms throughout the study. A total of 179 patients with URTI entered and completed the study. Each patient was randomly assigned to study medication, either Kan Jang® capsules (94 patients; males/females 47/47; mean age: 35.62 ± 1.314 years) or placebo capsules (85 patients; males/females 41/44; mean age: 34.08 ± 1.52 years). The patients' demographic and clinical data were compared to ensure comparability between the verum and placebo groups, and no significant differences were found (Table 2). The severity of the seven URTI symptoms (sore throat, runny nose, nasal congestion, hoarseness, cough, headache, fatigue) at the beginning of the study was comparable in the two treatment groups showing no significant differences (Table 2). General hematological analysis showed no significant differences between the two groups (Table 1 in Supplement 5).

### 3.2 Analysis of efficacy

#### 3.2.1 Efficacy outcomes

The primary efficacy outcome measure was the duration of the acute phase of disease assessed as the number of sick leave days at home (Fig. 2). Fig. 2a shows that more patients terminated sick leave at home in the Kan Jang® group during the first four days compared with that in the placebo group. The time to major improvement, defined as the number of sick leave days at home (Fig. 2c), correlated with the patients' recovery rate (percentage of recovered patients/time of treatment; Fig. 2b), which was significantly higher in the Kan Jang® group than in the placebo group on days 2, 3, and 4. On treatment day 3, 75% of the patients in the Kan Jang® group and 55% in the placebo group had recovered (significance level p = 0.0008, OR 3.1429, z-statistic 3.352; statistical significance: 95% confidence; Supplement 3). Sick leaves at home were terminated by 50% of the patients in 1.7 days in the Kan Jang® group and 2.5 days in the placebo group (Fig. 2b) (2.38 vs. 3.02 days, respectively; p = 0.0053), whereas the median number of days at home was 3 days in the placebo group and 2 days in the Kan Jang® group (Fig. 2c and Supplement 3) (median ratio A/B, 1.5 [95% CI: 1.113–2.021], hazard ratio A/B, 0.4954 [95% CI: 0.3263–0.7520]). The mean duration at home was shorter in the Kan Jang® group than in the placebo group (2.38 vs. 3.02 days, p = 0.0053) (Fig. 2d).

**Fig. 3:**
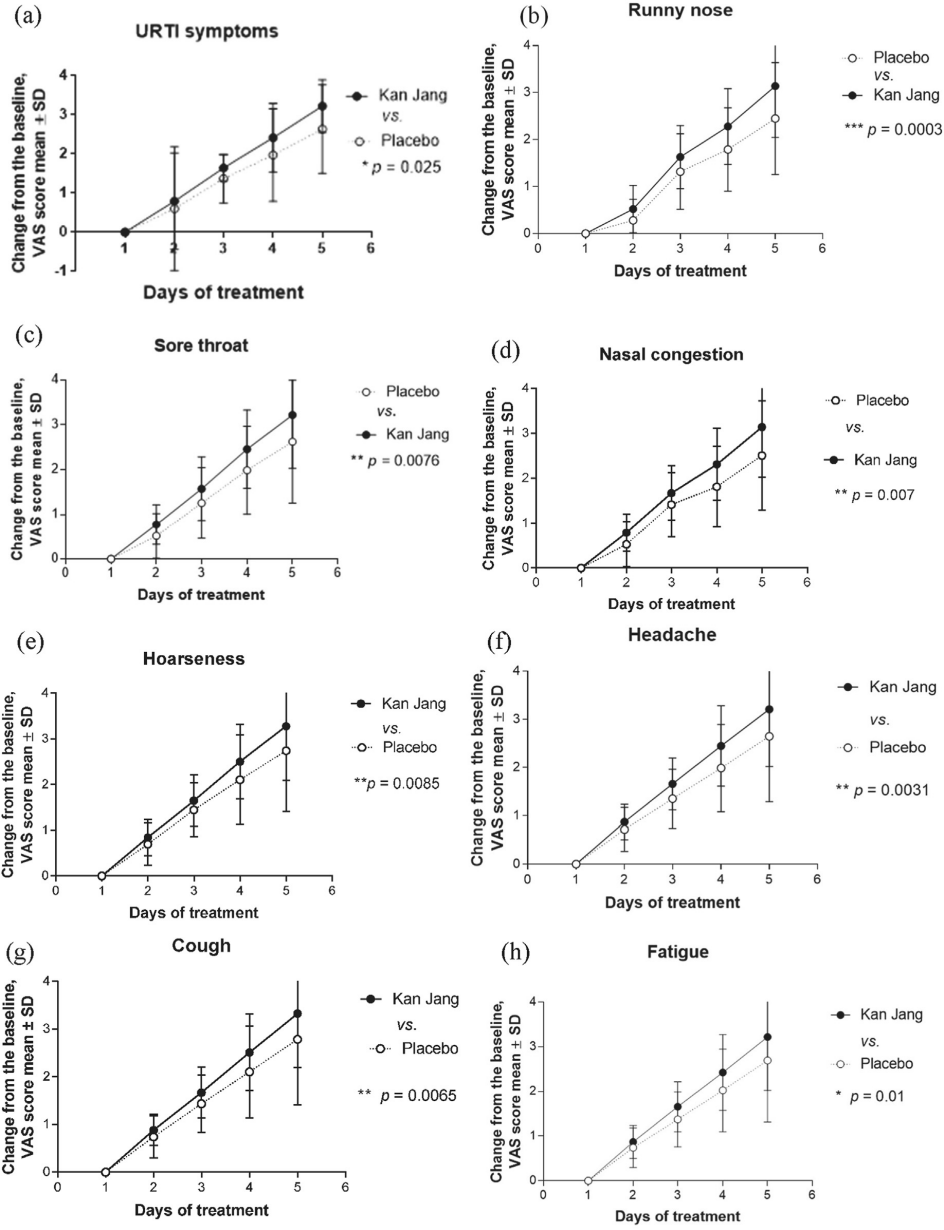
Effects of Kan Jang® (Group B) and placebo (Group A) on inflammatory symptoms, as assessed by the change in the total VAS score from baseline (mean ± SD) during the 5 days of treatment. Statistically significant (p = 0.025, two-way ANOVA) interaction effect between treatment groups and response over time showed a significant difference between Kan Jang (group B) vs. placebo (Group A). Y-axis: reduction (change from baseline) in the total symptom VAS score.

The secondary efficacy outcome was the relief of URTI symptoms—sore throat, nasal obstruction, nasal discharge, cough, hoarseness, headache, and general malaise. These symptoms were self-assessed by questionnaires using 10-point-scale scores and calculated as the mean TSS. Fig. 3 shows the improvement in URTI symptoms starting from the second day of treatment until the 5-day treatment. Kan Jang® significantly attenuated URTI symptoms relative to those by placebo. The interaction effect was significantly different over time between the two groups (p < 0.05). The diagram shows the reduction (change from the baseline) of the total VAS scores for all symptoms after 5 days in both groups. A significant difference between the groups was observed from the second day of treatment, and this difference increased gradually over the following days (Fig. 3, Supplement 3**).**

**Fig. 4:**
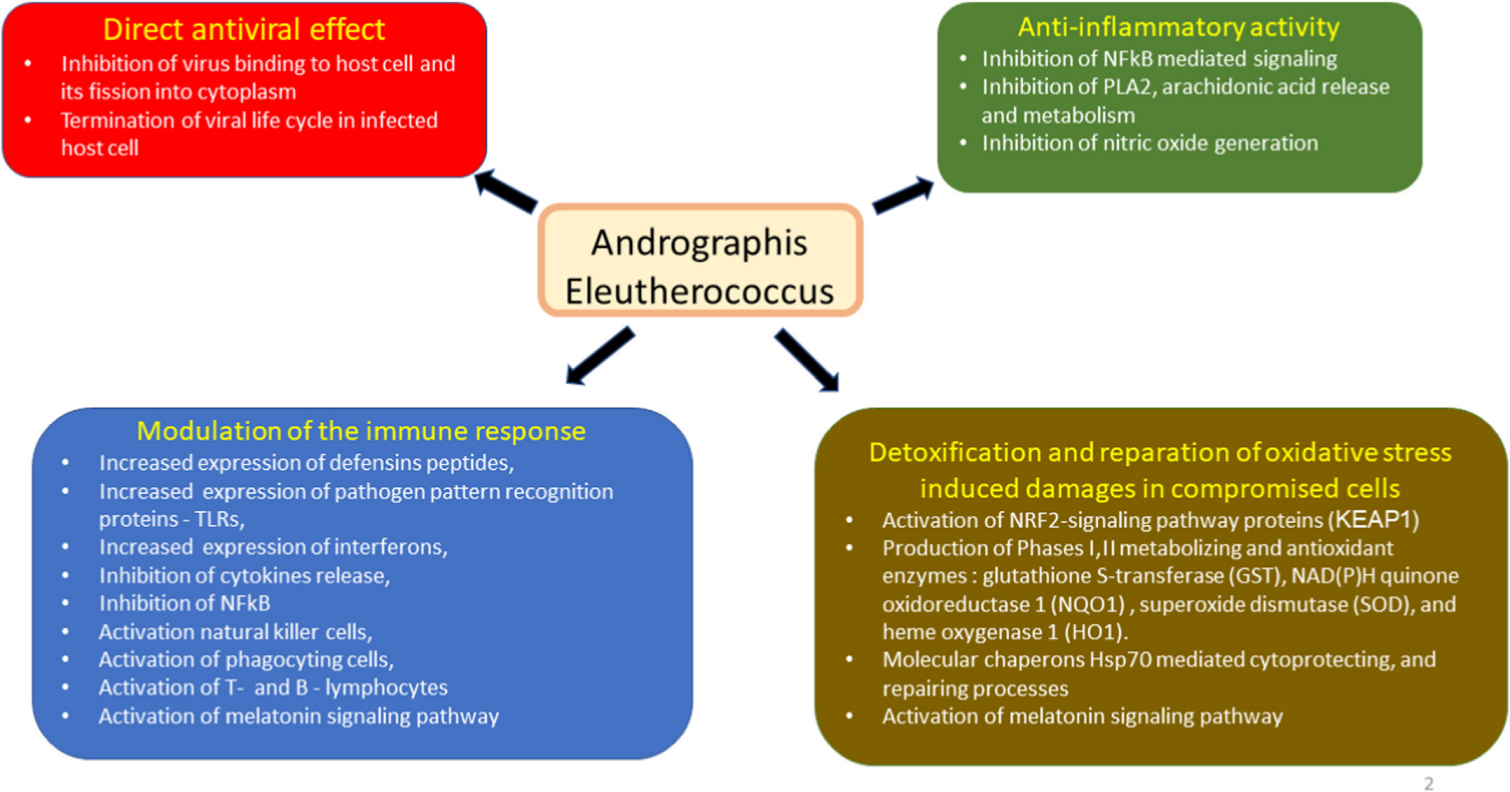
Schematic diagram of reported effects of Andrographis, Eleutherococcus or their combination Kan Jang elucidated in animal, and cell culture models: (i) modulatory effects on immune response (blue block), (ii) anti-inflammatory activity (green bock), (iii) detoxification and repair of oxidative stress-induced damage in compromised cells (brown block), and (iv) direct antiviral effect via infraction with viral docking or replication (red block).

Statistical significance in intergroup differences was observed in all of seven URTI symptoms, i.e., sore throat, nasal obstruction, nasal discharge, cough, hoarseness, headache, and general malaise (Fig. 4b–h). On treatment day 2, significant differences in all symptoms were observed between the Kan Jang and placebo groups (Supplement 3). This trend increased gradually during the recovery of the patients.

Blood analysis was performed to assess the inflammatory response from the URTI. A comparison between the Kan Jang® and placebo group at the end of the study showed a significant difference in the blood parameters (Table 3). The intergroup analysis showed that the normalizing effect on WBC and ESR was significantly better for Kan Jang® than for placebo (p = 0.007 and 0.0258, respectively).

### 3.3 Safety results

The treatment with Kan Jang® was well tolerated. The only common adverse reaction was mild pruritus observed in 10 patients (four in the Kan Jang® group B and six in the placebo group A, OR B/A = 0.5852, z-statistics = 0.807, p = 0.4194). No serious adverse reactions, such as allergic reactions (e.g., urticaria, angioedema, paresthesia, anaphylactic reactions, or rush), were observed (Supplement 4).

## 4 Discussion

The progression and recovery of URTI diseases include four overlapping phases of the acute inflammatory response to the pathogen: initiation, transition, resolution, and return to tissue homeostasis.^12^ Acute inflammation is a protective, defensive, and self-limited response for eliminating infectious pathogens and repairing damaged tissues to reestablish homeostasis.^12^ This highly coordinated process involves various innate immune cells, pro-inflammatory and pro-resolving mediators, and mechanisms.^12,^^13^ Consequently, effective treatment of URTI requires pharmaceutical corrections of many components of the innate, adaptive immune system, phases I–III metabolizing enzymes of detoxifying and repair systems, and the viral life cycle and proliferation. Successful treatment can be achieved by multitarget pharmaceutical intervention of herbal preparations that have polyvalent and pleiotropic actions on host defense systems, such as *A. paniculata* and *E. senticosus* extracts and their fixed combination, Kan Jang®, which are known to exhibit antiviral, immunomodulatory, and anti-inflammatory effects (Fig. 4; for details see Table 1, Table 2, Table 3, Table 4 in Supplement 6, adapted from Panossian and Brendler 2020^5^).^5^^,^^6^^,^^14^, ^15^, ^16^, ^17^, ^18^, ^19^, ^20^, ^21^, ^22^, ^23^, ^24^, ^25^, ^26^, ^27^

**Table 4:** Summary of Kan Jang® (*A. paniculata* and *E. senticosus* extracts) treatment outcomes in previous clinical studies.

First author name	Melchior	Melchior	Gabrielian	Kulichenko	Kulichenko	Spasov	Narimanyan
Year	2000	2000	2002	2003	2003	2004	2012
Diagnosis	URTI	URTI	URTI, sinusitis	Influenza	Influenza	influenza	URTI
Type of study	R-PC-DB	R-PC-DB	PC-DB	R-PC-OL pilot	R-PC-OL	R-C-DB	R-PC-DB prospective
Number of patients verum/control	23/23	89/90	95/90	71/469	35/31	53/41/39 children	94/85
Negative control	Placebo	Placebo	Placebo	Placebo	Placebo		Placebo
Positive control				Amantadine, paracetamol and vit. C	Amantadine, paracetamol and vit. C	Immunal (Echinacea extract)	
Daily dose	12 tablets	12 Tablets	12 tablets	6 tablets	9 tablets	6 tablets	12 capsules
Daily dose of andrographolides, mg	60	60	60	30	45	30	60
Duration of treatment, days	5	5	5	3–5	5	10	5
Cough	⓪	⓿	⓿	⓪	⓿ dos	⓪	⓿
Sore throat	⓿	⓿	⓿	⓪	⓿ dos	⓪	⓿
Nasal secretion	⓪	⓪	⓿	⓪	⓿ dos	⓿	⓿
Nasal congestions	⓪	⓪	⓿	⓪	⓿	⓿	⓿
Hoarseness	⓪	⓪	⓿	⓪	⓿	⓪	⓿
Headache	⓪	⓪	⓿	⓿	⓿ dos	⓪	⓿
Muscle soreness	⓿	⓿	⓪	⓿	⓪		
Eye discharge	⓪	⓪	⓪	⓿	⓿ dos	⓪	
Temperature	⓪	⓪	⓪	⓿	⓪	⓪	⓿
Malaise/fatigue			⓿	⓪	⓪		⓿
**Total symptoms**	⓪	⓿	⓿				⓿
Duration of sick leave at home, days				⓿ 7.2 vs. 9.8	⓿ 7.2 vs. 9.8		⓿ 2.4 vs. 3.0
Number of patients at home, median ratio							⓿ 1.5
Post-influenza Complications					⓿		
Blood hematology						⓪	⓿
IL-8, IgA, IgG						⓪	

Statistical significance of differences between Kan Jang® and placebo: ⓪ –p > 0.05, ⓿ –p < 0.05, ⓿ - not assessed.

In this context, it is not surprising that Kan Jang® is more effective compared with its individual ingredients (*A. paniculata* and *E. senticosus* extracts), apparently due to synergistic interactions between molecular networks.^17^^,^^18^ Among 250 deregulated genes, 111 were unique for the Kan Jang® combination and were not affected by either the *A. paniculata* or *E. senticosus* extract alone. Specific and predictable to Kan Jang® are the effects on canonical pathways and networks associated with infectious and chronic inflammatory disorders (Fig. 1 in Supplement 6).^18^ This is in line with the results of a meta-analysis of two studies that compared the clinical efficacy of Kang and Andrographis (Calm Cold).^16^ Furthermore, the antiviral activity of the Kan Jang combinations of Andrographis and Eleutherococcus was greater than expected in a recent study that demonstrated direct antiviral activity of Andrographis, Eleutherococcus, and their combination Kan Jang® against the coronavirus.^17^

Five pilot, interventional, and prospective studies have been conducted with Kan Jang® tablets in a total of 460 patients with acute viral respiratory infections.^7^, ^8^, ^9^, ^10^, ^11^ The aim was to determine the most effective dose and treatment schedule and identify which of the inflammatory symptoms are most significantly affected by the Kan Jang® combination. For this purpose, variations in the study populations (adults^7^, ^8^, ^9^, ^10^ vs. children^11^), the severity of infections (common cold^7^, ^8^, ^9^ vs*.* influenza^10^^,^^11^), study design (preventive^7^ vs. curative treatment^8^, ^9^, ^10^, ^11^), active doses (two tablets^7^^,^^9^ vs. six tablets^10^^,^^11^ and nine tablets^10^ vs. twelve tablets^8^), treatment duration (3–5 days^10^ vs. 5 days,^8^^,^^9^ 10 days,^11^ and 3 months^7^), control groups (placebo^7^, ^8^, ^9^^,^ vs*.* standard treatment^11^ vs. positive control^10^^,^^11^), and efficacy outcome measures were investigated. The results of these studies are summarized in Table 4.

In these studies, Kan Jang® tablets alleviated various symptoms of URTI^8^^,^^9^ and influenza,^10^^,^^11^ including cough, sore throat, nasal secretion and secretion, hoarseness, headache, muscle soreness, eye discharge, body temperature, and malaise/fatigue. Table 5 shows the associations between the various symptoms of the common cold^28^ and the mediators of inflammation involved in activating adaptive and innate immune systems in response to infectious challenges. Kan Jang supports this adaptive immune response, apparently both due to Eleutherococcus and Andrographis, which stimulate the immune system via different mechanisms (Fig. 4 and Supplement 6), including the activation of Hsp70 expression. Kan Jang activates the adaptive and innate immune system, increases the heat shock protein Hsp70 in the blood (resulting in accelerated reparation and disposal of damaged or defective proteins), and protects cells from further heat- and infection-induced damage. It can be concluded that the immune-supporting effect of Kan Jang accelerates the recovery of patients and relieves the inflammatory symptoms earlier than andrographolide alone.

**Table 5:** Mediators of inflammation involved in the inflammatory symptoms most affected by Kan Jang (adapted from Panossian & Wikman, 2012).^17^

Cell	Mediator	Target tissue, cells	Effect	Inflammatory symptoms
**must cell, eosinophils,**	histamine PAF leukotrienes	Vessels	Vascular permeability increases Vasodilatation-blood stream decreases Leukocyte extravasation increases	Sneezing edema reddening's/warming
		Neurons	Activation of nociceptors	Pain
		Airway epithelium	Airway sensory nerve endings	Cough
**macrophages**	IL-1, IL-6, TNFa,	Brain		fatigue, headache, malaise, anorexia sleep disturbance
		Hypothalamus	Interact with the vagus nerve endings to signal the temperature control center	fever
	prostaglandins	Skin blood vessels	Vasoconstriction	chilliness
		Skeletal muscle	Effects on peripheral pain receptors	muscle ache, pain
		Muscles	Catabolism	weight loss
	Leukotrienes	Fat tissue	Lipolysis	
		Neutrophils Monocytes	Chemotaxis, phagocytosis.	nasal discharge mucous secretion
	PAF	Bacteria, virus	Phagocytosis	
		Immune system	Immune defense	all symptoms
	prostaglandins bradykinin	Nerve endings in the airway	Pain mediated by the cranial nerves Supplying the nasopharynx and pharynx.	sore throat
**blood plasma globulins**	Bradykinin	large veins in the nasal epithelium	Vasodilation	nasal congestion

Overall, Kan Jang decreased total URTI symptom scores after five days of treatment. However, direct evidence supporting an accelerated recovery rate during the early phase of progression of URTI symptoms is not well documented in the scientific literature. An unpublished pilot multicenter study in Sweden (Table 4, Melchior et al., 2009, unpublished in-house report^29^) showed that the most common illness symptoms declined faster in the Kan Jang® group than in the placebo group. We also found that the number of days of sick leave was significantly higher in the placebo group (1.7 days) than in the Kan Jang® group (0.5 days).

Our study provides the first evidence that by day 2 of treatment with Kan Jang®, all seven URTI symptoms improve significantly (Fig. 3 and Supplement 3). On day 3 of treatment, 62% of the patients in the Kan Jang® group had recovered compared with 43% of the patients in the placebo group (Fig. 2 and Supplement 3). Furthermore, the Kan Jang®-accelerated recovery of URTI symptoms is associated with anti-inflammatory effects, observed as significant normalization of the WBC count and ESR among those patients treated with Kan Jang® (Table 3). These results are consistent with those of previous studies that investigated Kan Jang® for the treatment of inflammatory symptoms.^7^, ^8^, ^9^, ^10^, ^11^

These results easily translate into a significant economic value because the URTI costs for a small business can be reduced by 75%. For a small business with 20 employees, an average absence due to illness of 5.5% (242 days), out of which ≥25% are due to common colds, i.e., 1.4% (60 days) and a cost of € 200 for losing one workday, translates into € 9000 per year that could be saved if all employees started taking Kan Jang® capsules as soon as they felt URTI symptoms. This figure is a conservative estimate as to the prevention of resulting bacterial infections that could follow untreated infections is not shown in the statistics above, and thus not included in the sum of € 9000 per year, and absenteeism due to a sick child and presenteeism (present at work while sick and not being able to produce fully) are not included either.

According to the Global Burden Disease Study 2013 published in *The Lancet* in 2015, 18.8 billion people suffered from URTIs in 2013.^30^ Hence, URTI-associated health complications have become an increasing economic burden on society. Early intervention of Kan Jang® capsules in the acute phase of URTI is crucial for preventing further health complications and reducing sick leave days.

The limitations of this study include a relatively small sample size, which, however, has no impact on the statistical significance of the results obtained in this clinical trial. There was no limitation regarding the type of pathogens that induced URTI, including acute nasopharyngitis (common cold), sinusitis, pharyngitis, tonsillitis, laryngitis, and tracheitis, which are primarily induced by rhinoviruses. The pathogen of the symptoms of the upper respiratory tract infection has not been identified in every case because that was not the aim of our study. The clinical signs and their severity (particularly body temperature) needed to be the same in all patients recruited in the study. Patients with influenza and pneumonia were also excluded from the study.

However, based on the preclinical assessment of Kan Jang and its ingredients^14^, ^15^, ^16^ and recent publications,^5^^,^^6^^,^^17^ we suggest that Kan Jang might help increase the recovery rate of other acute and chronic respiratory diseases, including COVID-19.

Recently, these herbal preparations have also been recommended for the prevention of COVID-19 as well as for adjuvant therapy and recovery of COVID-19 patients.^5^^,^^6^ Furthermore, Thailand's health ministry approved using an Herba Andrographidis extract to treat the early stages of Covid-19 as a pilot program during a flareup in the coronavirus outbreak in Thailand.^31^^,^^32^ Further studies regarding Kan Jang/agaDA® in COVID-19 patients are needed to verify whether the recently observed *in vitro* direct antiviral effect of Kan Jang^17^ has clinical significance. Moreover, assessment of the efficacy of Kan-Jang® in mild COVID-19 is currently in progress.^33^

## 5 Conclusions

In this randomized, placebo-controlled, parallel-group, prospective, randomized phase II study, we demonstrated that Kan Jang® capsules significantly increased the recovery rate in the first days of interventions, which significantly reduced the number of days of sick leave due to illness. The WBC count and ESR in the blood were also decreased by Kan Jang® relative to those by placebo in URTIs. Compared with placebo, Kan Jang® significantly alleviated the severity of inflammatory symptoms. The treatment was well-tolerated, and no adverse events were observed. Kan Jang® medication is a suitable treatment in addition to standard therapy for reducing URTI symptoms and sick leave duration while also minimizing or eliminating the need for antibiotics.

## Author contributions

Mikael Narimanyan was responsible for project administration, methodology, investigation, formal analysis, validation, data curation, resources, writing original draft, and editing. Kristina Jamalyan and Anna Balyan conducted the study, ensured that the demands from the study itself, and the participants, could be met for study results, and tabulated the collected data. Anders Barth supervised the conduct of the study. Staffan Palm contributed to writing, review, and editing. Georg Wikman contributed to the conceptualization of the study, resources, funding acquisition, and reviewing of the manuscript. Alexander Panossian contributed to statistical analysis of the datasets, writing, and editing of the manuscript.

## Funding

This study was sponsored by the Swedish Herbal Institute in Vallberga, Sweden; Grant Nr 2011–3.

## Declaration of competing interest

Georg Wikman declares a conflict of interest because he is the president of SHI Research and Development AB. Staffan Palm, Panafarma Medical Consult AB, is an independent agreement contractor with the Swedish Herbal Institute. Alexander Panossian is self-employed at the research and development company Phytomed AB. He was the former Head of Research & Development at the Swedish Herbal Institute, Gothenburg, Sweden. He has no shares or financial interest in any pharmaceutical company. The other authors declare no conflicts of interest. The funder had no role in the design of the study; collection, analyses, or interpretation of data; writing of the manuscript, or decision to publish the results.
