# Supplementary figures and images for: Early intervention with Kan Jang® to treat upper-respiratory tract infections: A randomized, quadruple-blind study
Source: J Tradit Complement Med. 2021 Jun 11;11(6):552–62. doi: 10.1016/j.jtcme.2021.06.001 (PMC8572720; doi:10.1016/j.jtcme.2021.06.001)

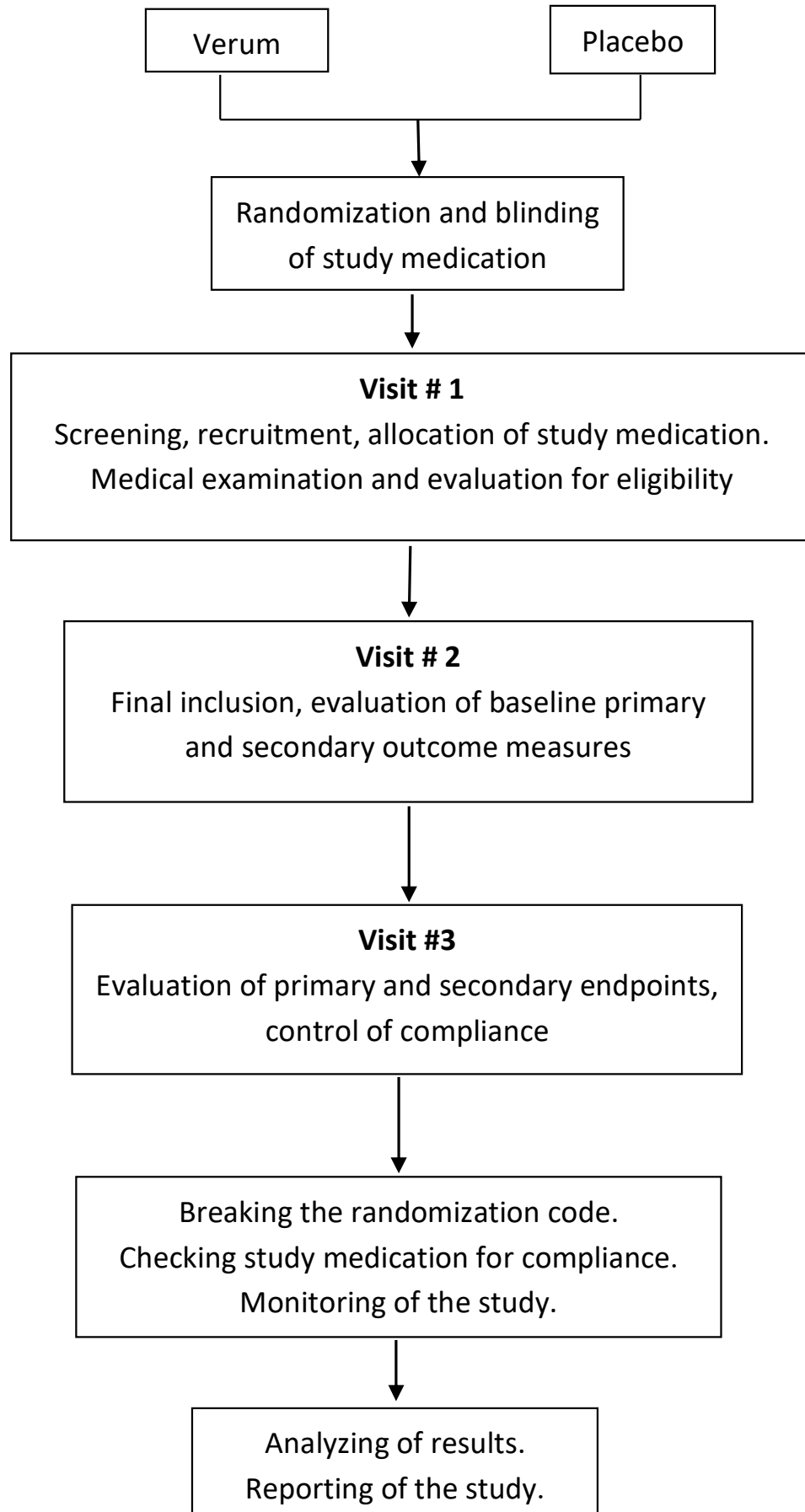

Supplement: Multimedia component 1 [file mmc1.pdf]
