# Supplementary material for: Early intervention with Kan Jang® to treat upper-respiratory tract infections: A randomized, quadruple-blind study
Source: J Tradit Complement Med. 2021 Jun 11;11(6):552–62. doi: 10.1016/j.jtcme.2021.06.001 (PMC8572720; doi:10.1016/j.jtcme.2021.06.001)
Supplement: Multimedia component 2 [file mmc2.pdf]

SUPPLEMENT 1

Name of the medicinal product

KanJang Andrographis® with fixed combination of Andrographis paniculata herb extract and Eleutherococcus senticosus root extract.

Pharmaceutical form

Capsules

Indication for use

Herbal Medicinal Product for the *“reduction in the severity and duration of symptoms of uncomplicated respiratory tract infections (common cold)”*.

Dosage

The recommended daily dose is 2 capsules 3 times per day for adults.

Quantitative Composition

**Substance**	**Quantity per one capsule, mg**
Active ingredients	
Herba Andrographidis standardised extract containing 66-110 mg of native dry extract	250
(DER 4,5-8,0 :1, extraction agent - ethanol* 70 %, V/V) corresponding to **10 mg** of the sum of andrographolisdes (andrographolide and deoxydidehydro-andrographolide) and 45-67% of maltodextrin**(adjuvant for adjustment)	
Radix Eleutherococci root extract, containing 11.4 mg of native dry extract (DER 17-30:1, extraction agent – ethanol 70%, V/V) , corresponding to 194-342 mg of herbal substance (Radix Eleutheorcocci, standardized for the content of eleutherosides B and E (0.4 mg/capsule) and 50% of maltodextrine** (adjuvant for adjustment)	22,8
Inactive ingredients	
Microcrystalline cellulose	70
Magnesium stearate	7.2
**Vegetable capsule**	75
**Total weight**	425 ± 21

Swedish Herbal Institute

R&D

Kan Jang Andrographis
Clinical study in Armenia

Product: **Kan Jang Andrographis**
Product category: Herbal medicinal product
Dosage form: capsules
Daily dose: **6 capsules size 1**
Batch number: 50145
Batch size: 5000 capsules

Composition

**Ingredients**	Ratio	Ratio native extract	1 capsule contains		
			herbal substance, mg	native extract, mg	dry extract, mg
Andrographis paniculata herb, dry extract batch nr: 1521114	3.9:1	6.7:1	1003.47	195.7<br>10mg andrographolides	249,0
Eleutherococcus senticosus, radix dry extract, batch nr:1521077	9,00:1	18.00:1	205.00	11,4	22,8
Microcrystalline cellulose, batch nr. 1960752					85,0
Magnesium stearate, batch nr. 1960738					7,2
Total					364,00
Capsules of plant origin size 1, colour: clear, weight – 76 mg					
Hypromellose – 100%, water content – 6%					
Filled capsules weight			440,00mg±33mg		

Prepared by (R&D): Susanna Panosyan Date:
Checked by (R&D): Alexander Panossian Date:

Swedish Herbal Institute

R&D

Kan Jang Andrographis
Clinical study in Armenia

Product: **Placebo**
Product category: Herbal medicinal product
Dosage form: capsules
Daily dose: **6 capsules size 1**
Batch number: 50137-1
Batch size: 4000 capsules

Composition

**Ingredients**	1 capsule contains, mg
Lactose monohydrate, batch nr. U2EX 417	205
Decaffeinated coffe, batch nr. U2EX 424	151
Magnesium stearate	8
Total	364
Capsules of plant origin size 1, colour: clear, weight – 76 mg	
Hypromellose – 100%, water content – 6%	
Filled capsules weight	440 mg±33 mg

Prepared by (R&D): Susanna Panosyan Date:
Checked by (R&D): Alexander Panossian Date:

Bulk

Svenska Örtmedicinska Institutet

Swedish Herbal Institute

Certificate of analysis

Capsule: Kan Jang Andrographis Batch no: 50.145 Doc.no: ANJC.

Extr. Andr. sicc 1521114 Bulk batch no: — Internal spec. no: SP.FV-

Extr. Eleuth sicc 1521077

Extr. ....

Parameter of analysis	Method	Reference	Result
Appearance	Occular	SP.FV-	
Smell	O.L	SP.FV-	
Total ash	Ph.Eur.Curr.Ed		6%
Ash, insoluble in HCl	Ph.Eur.Curr.Ed		1%
Loss on drying	Ph.Eur.Curr.Ed		3%
Identity (TLC)	SM-010	=ref	=ref
HPLC			
Eleuth B	SM-066		0,084 mg/del
Eleuth E	SM-066		0,119 mg/del
Σ Eleutherosides			0,203 mg/del
Andrographolide			8,15 mg/del
DDD-andr.			1,50 mg/del
Σ Andr + DDD-andr			9,65 mg/del
Neoandr.			3,44 mg/del
Weight including capsule	Ph.Eur.Curr.Ed	440 ± 33 mg	418 ± 3 mg
Weight without capsule	-	364 ± 27 mg	342 ± 3 mg
Diameter	-		6,9 ± 0,1 mm
Length	-		19,3 ± 0,1 mm
Disintegration	Ph.Eur.Curr.Ed (SM-032)	≤ 30 min	< 30 min
		Reference	Released
Microbial contamination	Ph.Eur.Curr.Ed	Ph.Eur.Cat B	

Date of analysis: 111028 Date of release: 2011 11 14

Analyst, sign: NO/ AN QP-responsible: [illegible]

Comments:	Manufacturing date:
	Expiry date (two years):

Svenska Örtmedicinska Institutet
Swedish Herbal Institute

Kan Jang Andrographis capsules
HPTLC
50 145

Method: SM-010

VIS

Ref 3400036 | Test 50145

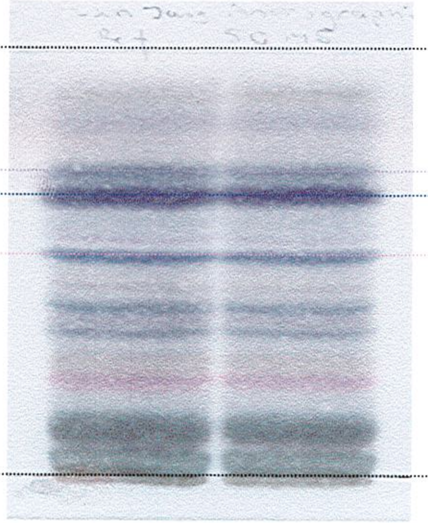

DDD-and
R_f: 0,71
Andr
R_f: 0,66
Neo-and
R_f: 0,51

254nm

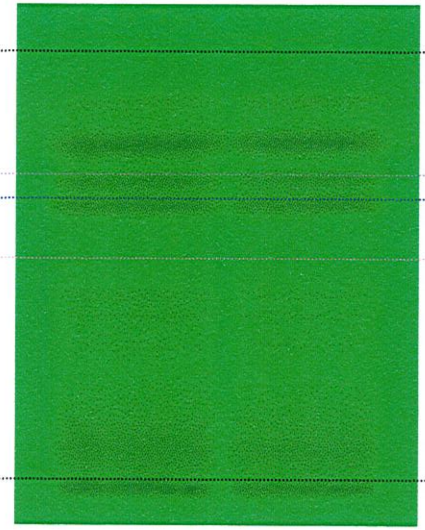

366nm

Front

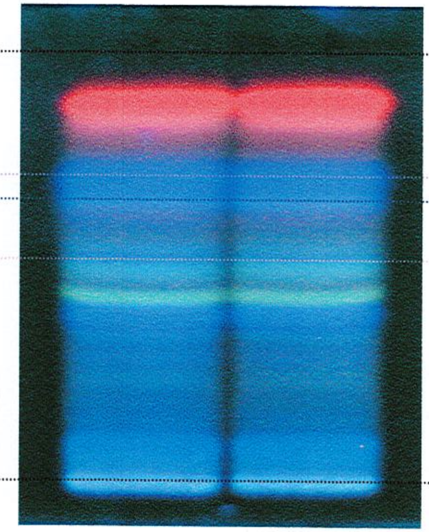

Start

Absorbent: Silica gel 60 F_{254} precoated HPTLC plates
Solvent system: Etylacetate : methanol : water (38 : 7 : 4)
Spray reagents: vanillin-sulphuric acid solution

Sign: Matilda

Date: 2011-11-02

Empower 2 software

Processing Method Andrographis

Start Date 2011-10-28 10:09:49 CEST

SampleName KJ kaps 50145

Name Andrographolide, Deoxy-didehydroandrographolide, Neoandrographolide

Current Date 2011-10-28

Software Empower 2 Software Build 215[illegible]

Column no.: Acquity UPLC BEH C18 1.7[illegible]

01773000715504

Channel 205 nm, 223 nm, 254 nm

Run Time 5.00 Minutes

Tbl. weight: 342 (418) mg

Component Results
Name: Andrographolide

	SampleName	SampleWeight	RT	Area	Height	Name	Percent_amount
1	KJ kaps 50145	3.12880	1.974	502517	316756	Andrographolide	2.384
2	KJ kaps 50145	3.12880	1.975	502858	317162	Andrographolide	2.385
3	KJ kaps 50145	3.12880	1.975	502143	317438	Andrographolide	2.382
Mean							2.383
Std. Dev.							0.002
% RSD							0.07

Component Results
Name: Neoandrographolide

	SampleName	SampleWeight	RT	Area	Height	Name	Percent_amount
1	KJ kaps 50145	3.12880	2.931	179403	167106	Neoandrographolide	1.009
2	KJ kaps 50145	3.12880	2.932	179028	166937	Neoandrographolide	1.007
3	KJ kaps 50145	3.12880	2.933	178994	166954	Neoandrographolide	1.007
Mean							1.007
Std. Dev.							0.001
% RSD							0.13

Component Results
Name: Deoxy-didehydroandrographolide

	SampleName	SampleWeight	RT	Area	Height	Name	Percent_amount
1	KJ kaps 50145	3.12880	3.201	73898	54169	Deoxy-didehydroandrographolide	0.439
2	KJ kaps 50145	3.12880	3.202	73800	54106	Deoxy-didehydroandrographolide	0.438
3	KJ kaps 50145	3.12880	3.202	74005	54232	Deoxy-didehydroandrographolide	0.439
Mean							0.439
Std. Dev.							0.001
% RSD							0.14

**Results**	**mg/tbl**	**calculated**	**%found**
**Andrographolide:**	8,15 mg/tbl	8,38 mg/tbl	
**Neoandrographolide:**	3,44 mg/tbl	3,70 mg/tbl	
**Deoxy-11,12-didehydroandrographolide:**	1,50 mg/tbl	1,59 mg/tbl	
**Total :**	13,09 mg/tbl	13,67 mg/tbl	96%

Sign.: W

Chromatogram

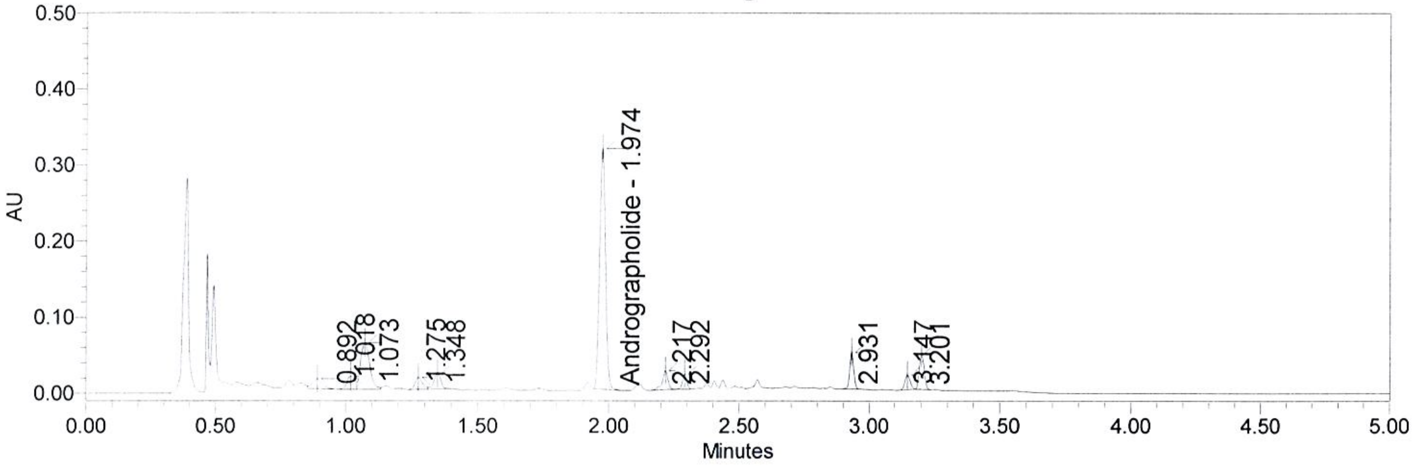

SampleName KJ kaps 50145; Vial 1:A,1; Injection 1; Date Acquired 2011-10-28 10:10:40 CEST; Channel Name 223 nm

Chromatogram

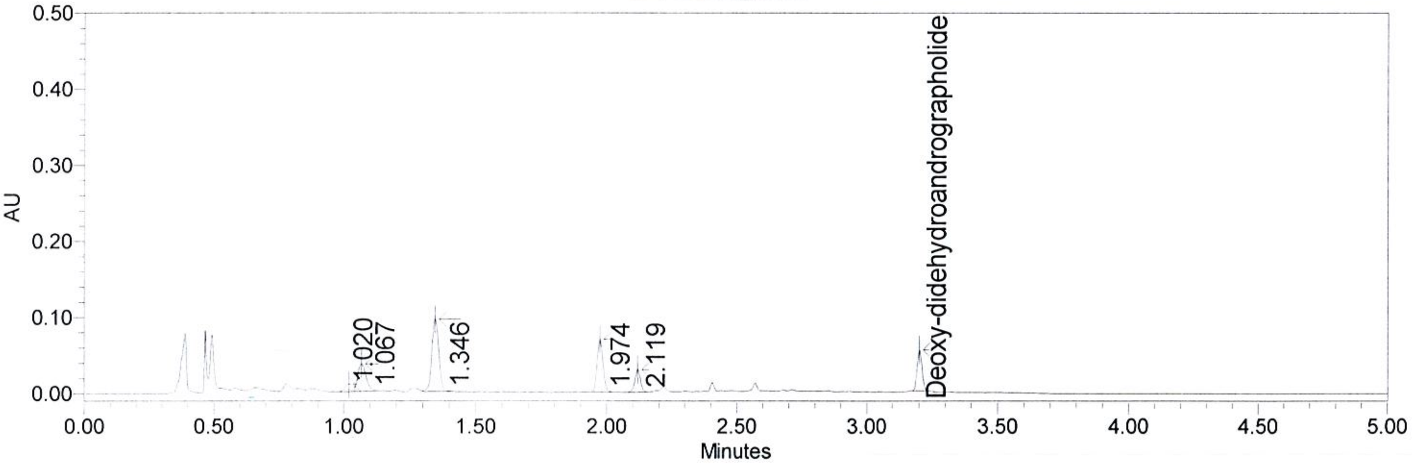

SampleName KJ kaps 50145; Vial 1:A,1; Injection 1; Date Acquired 2011-10-28 10:10:40 CEST; Channel Name 254 nm

Chromatogram

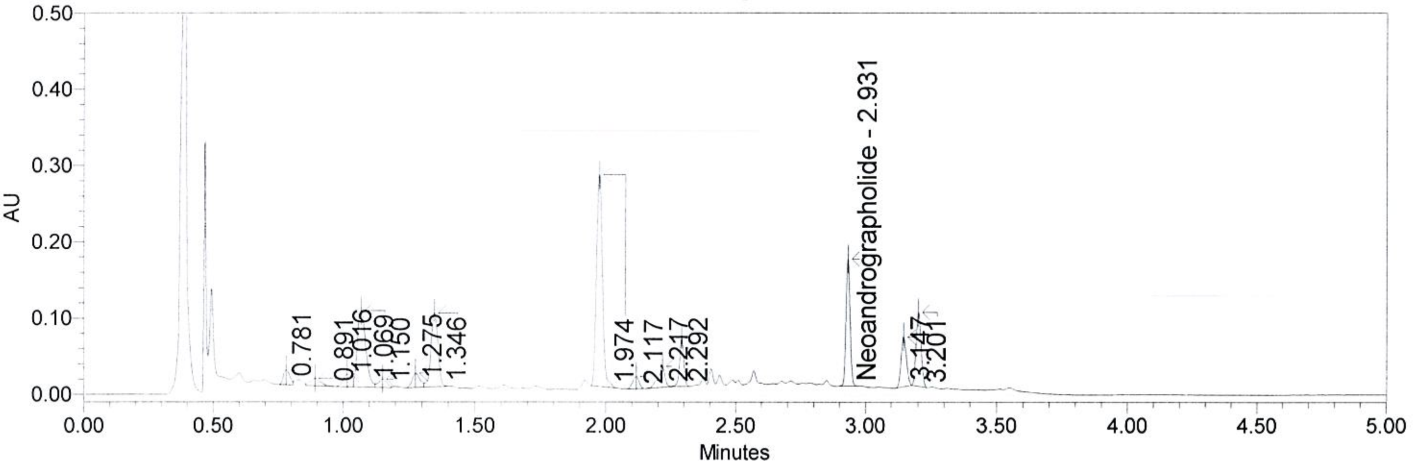

SampleName KJ kaps 50145; Vial 1:A,1; Injection 1; Date Acquired 2011-10-28 10:10:40 CEST; Channel Name 205 nm

Sign.:

Empower2 software

Current Date 2011-11-01
Processing Method Acanthopanax_KJT
Start Date 2011-10-31 10:28:24 CET
Tbl. weight: 342 (418) mg

SampleName KJ kaps 50145

Eleutherosid B
Eleutherosid E

Software Empower 2 Software Build 215
Column no.: 937031
Run Time 35.00 Minutes
Channel PDA_210.0nm, PDA_220.0nm
Tablet_Weight
System Name HPLC

Name: Eleutherosid B

	SampleName	Injection Volume (ul)	SampleWeight	RT	Area	Height	Name	Procent_Amount
1	KJ kaps 50145	20.00	606.00000	6.898	777314	50588	Eleutherosid B	0.0247
2	KJ kaps 50145	20.00	606.00000	7.548	774718	40082	Eleutherosid B	0.0246
3	KJ kaps 50145	20.00	606.00000	7.550	786407	43669	Eleutherosid B	0.0250
Mean								0.0248
Std. Dev.								0.0002
% RSD								0.78

Name: Eleutherosid E

	SampleName	Injection Volume (ul)	SampleWeight	RT	Area	Height	Name	Procent_Amount
1	KJ kaps 50145	20.00	606.00000	20.579	1147289	80158	Eleutherosid E	0.0353
2	KJ kaps 50145	20.00	606.00000	21.273	1144666	74949	Eleutherosid E	0.0353
3	KJ kaps 50145	20.00	606.00000	21.279	1153406	72314	Eleutherosid E	0.0355
Mean								0.0354
Std. Dev.								0.0001
% RSD								0.39

Results	***mg/tablet***	***calculated***	***% found***
Eleutherosid B:	0,085 mg/tbl	0,089	96 %
Eleutherosid E:	0,121 mg/tbl	0,114	106 %
Eleutherosides:	0,206 mg/tbl	0,203	101 %

Chromatogram

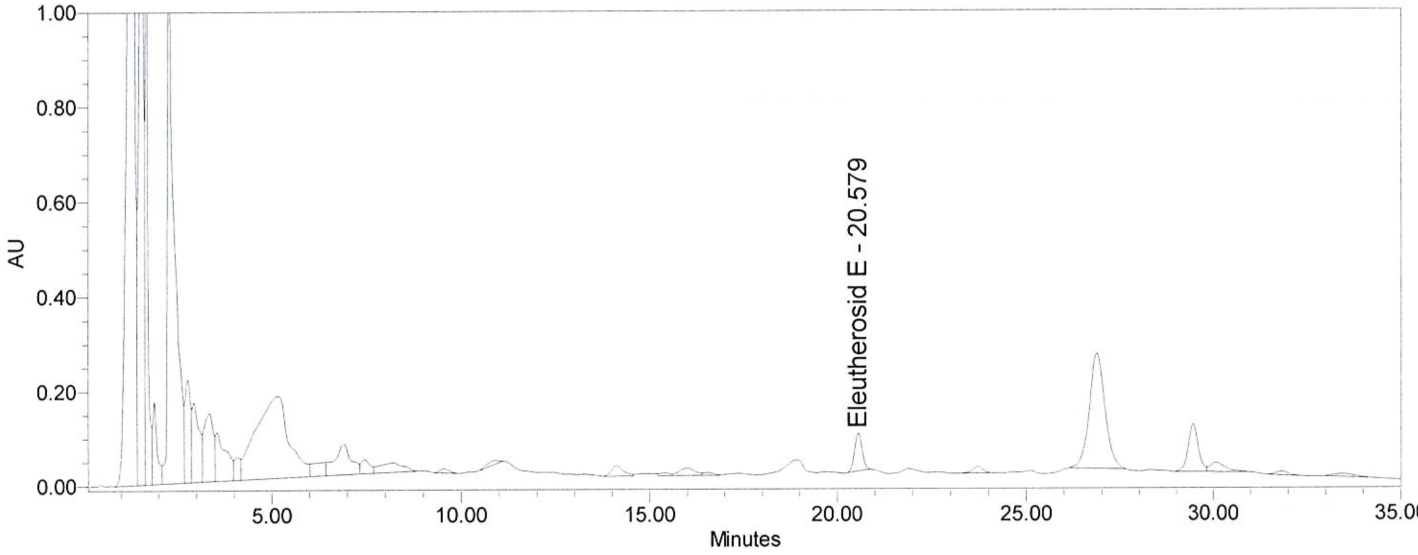

SampleName KJ kaps 50145; Vial 1; Date Acquired 2011-10-31 12:02:06 CET; Channel Name PDA_210.0nm

Sig.: [signature]

Chromatogram

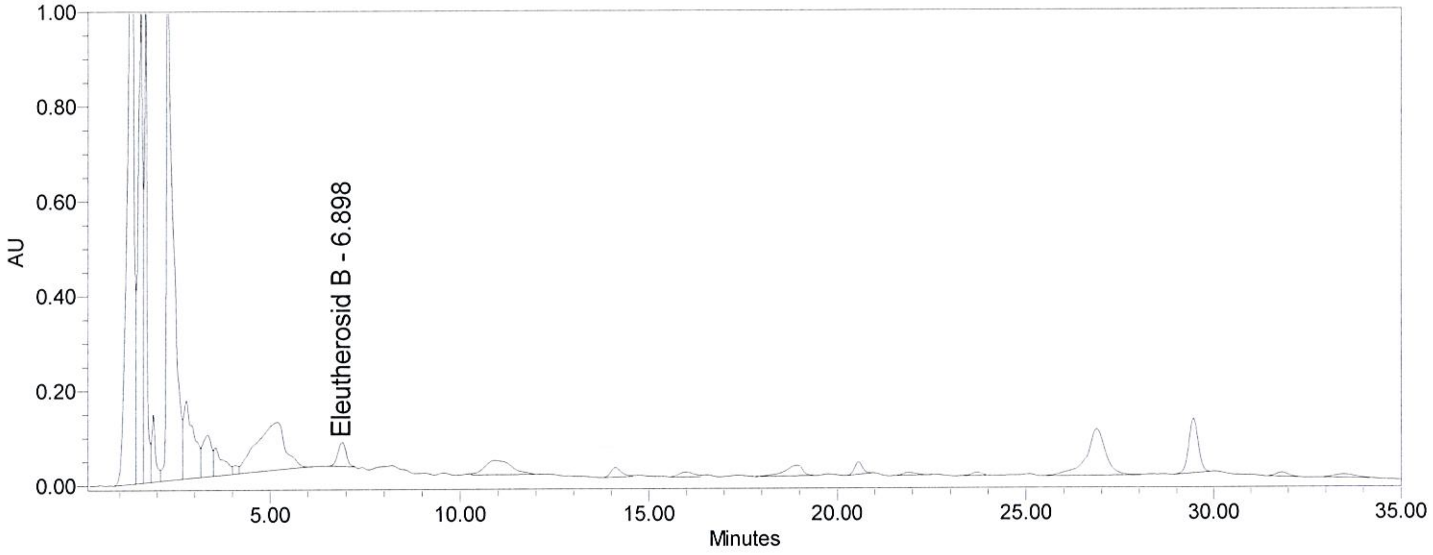

SampleName KJ kaps 50145; Vial 1; Date Acquired 2011-10-31 12:02:06 CET; Channel Name PDA_220.0nm

Chromatogram

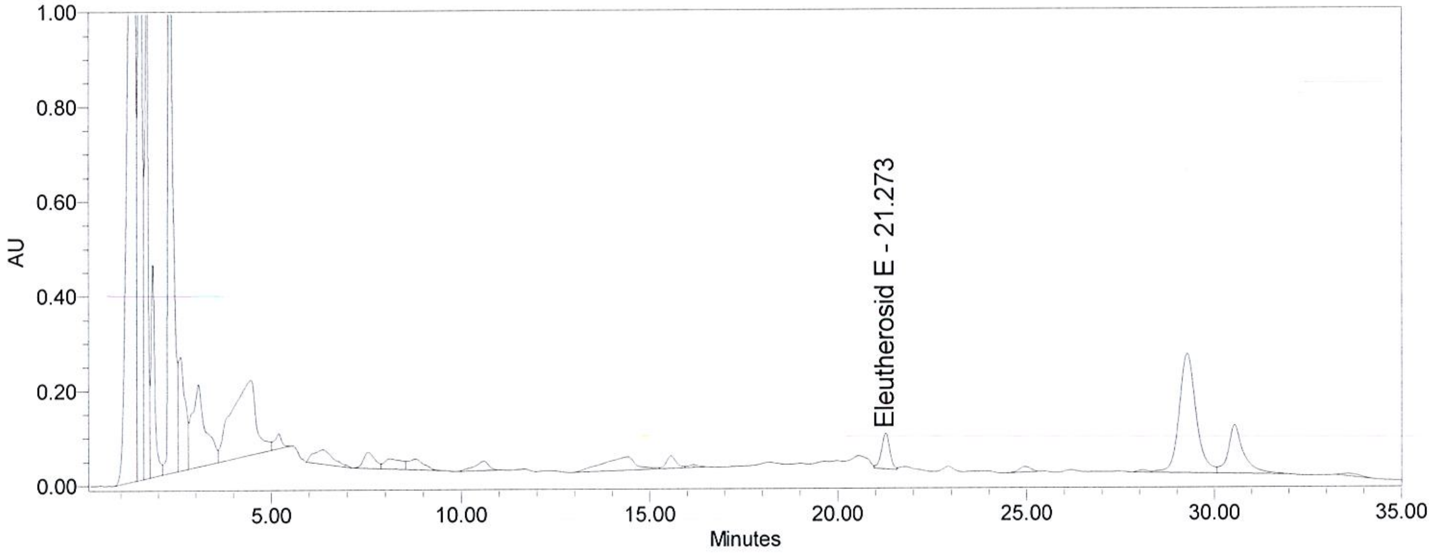

SampleName KJ kaps 50145; Vial 1; Date Acquired 2011-10-31 19:06:22 CET; Channel Name PDA_210.0nm

Sig.: [illegible]

Empower 2 software

SampleName KJ kaps 50145

Eleutherosid B
Eleutherosid E

Current Date 2011-11-07
Processing Method Acanthopanax_KJT
Start Date 2011-11-01 13:30:57 CET

Software Empower 2 Software Build 215[illegible]
Column no.: 937031
Run Time 35.00 Minutes
Channel PDA_210.0nm, PDA_220.0nm
Tablet_Weight 342 (418) mg
System Name HPLC

Name: Eleutherosid B

	SampleName	Injection Volume (ul)	SampleWeight	RT	Area	Height	Name	Procent_Amount
1	KJ kaps 50145	20.00	606.00000	7.062	769506	47784	Eleutherosid B	0.0245
2	KJ kaps 50145	20.00	606.00000	7.431	770998	41823	Eleutherosid B	0.0245
3	KJ kaps 50145	20.00	606.00000	7.455	777146	42652	Eleutherosid B	0.0247
Mean								0.0246
Std. Dev.								0.0001
% RSD								0.52

Name: Eleutherosid E

	SampleName	Injection Volume (ul)	SampleWeight	RT	Area	Height	Name	Procent_Amount
1	KJ kaps 50145	20.00	606.00000	21.107	1130714	72690	Eleutherosid E	0.0348
2	KJ kaps 50145	20.00	606.00000	21.207	1126124	73673	Eleutherosid E	0.0347
3	KJ kaps 50145	20.00	606.00000	21.223	1125963	73463	Eleutherosid E	0.0347
Mean								0.0347
Std. Dev.								0.0001
% RSD								0.24

Results	*mg/tablet*	*calculated*	*% found*
Eleutherosid B:	0,084 mg/tbl	0,089	94%
Eleutherosid E:	0,119 mg/tbl	0,114	104%
Eleutherosides:	0,203 mg/tbl	0,203	100%

Chromatogram

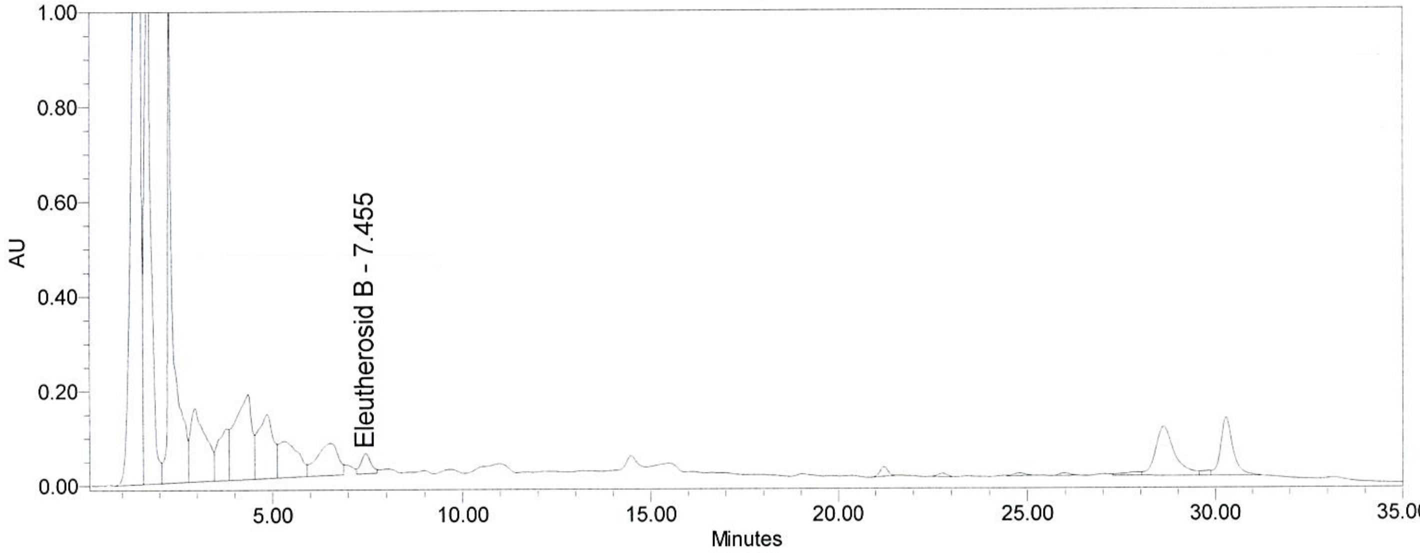

SampleName KJ kaps 50145; Vial 1; Date Acquired 2011-11-02 17:01:52 CET; Channel Name PDA_220.0nm

Sig.: [signature]

Chromatogram

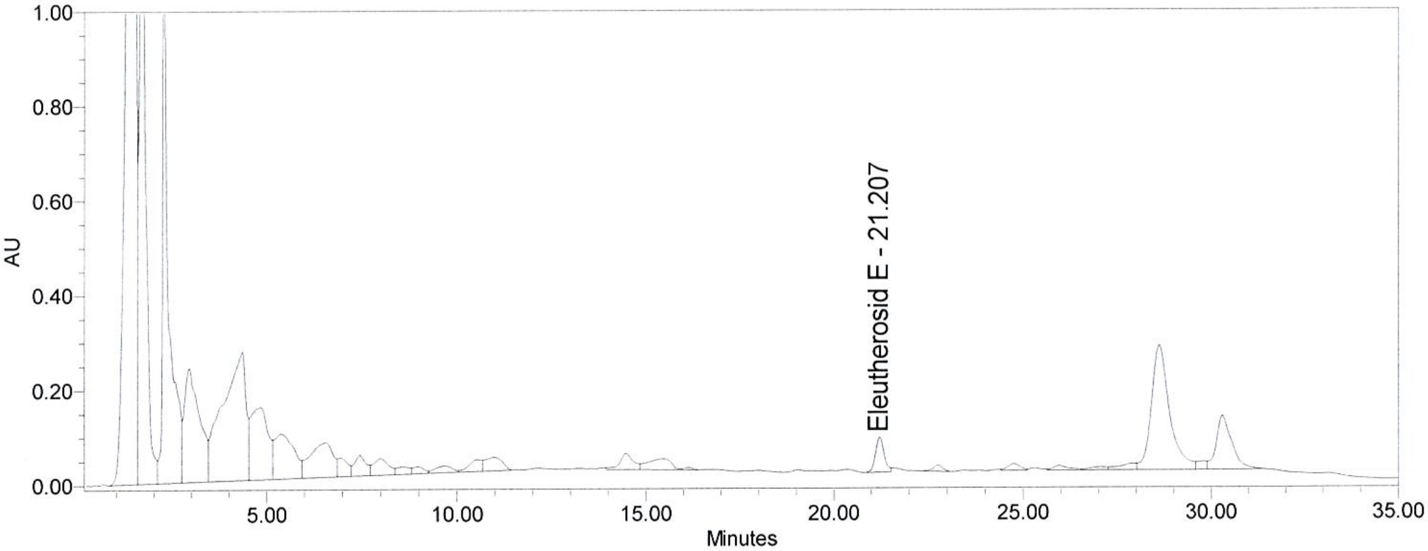

SampleName KJ kaps 50145; Vial 1; Date Acquired 2011-11-02 17:01:52 CET; Channel Name PDA_210.0nm

Chromatogram

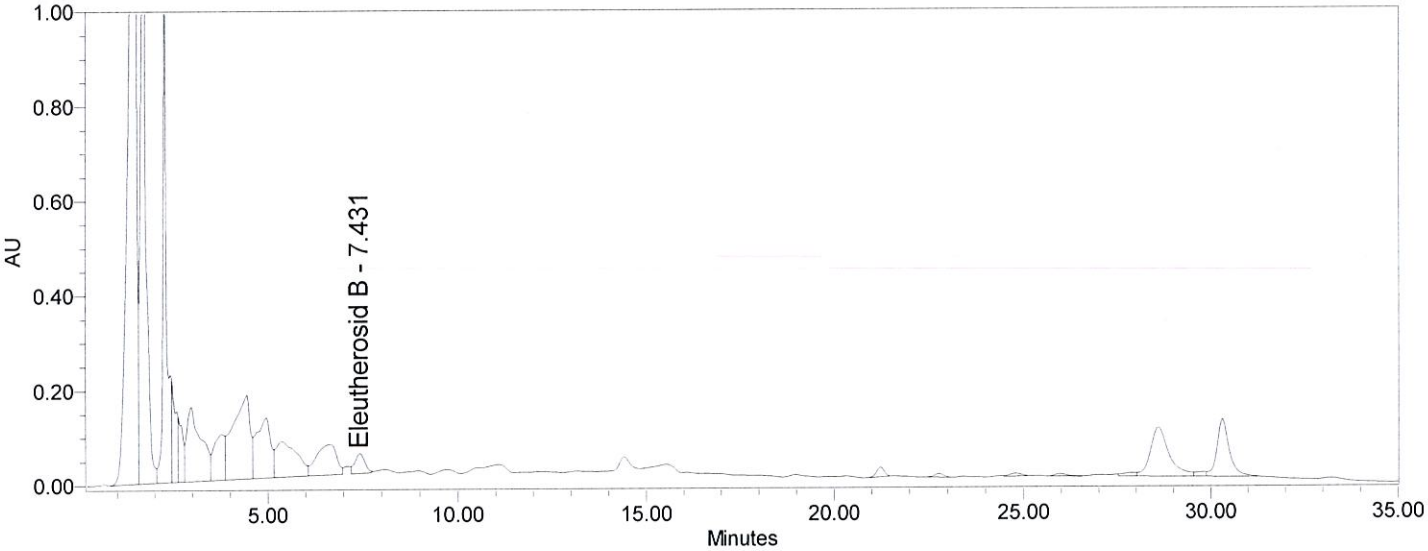

SampleName KJ kaps 50145; Vial 1; Date Acquired 2011-11-02 15:29:28 CET; Channel Name PDA_220.0nm

Sig.: [illegible]

Blister

Svenska Örtmedicinska Institutet

Swedish Herbal Institute

Certificate of analysis

Capsule: Kan Jang Andrographis **Batch no:** 50145

Extr. ....

Extr. ....

Extr. ....

Bulk batch no:.....................

Doc.no: ANJC.

Internal spec. no: SP.FV-

Parameter of analysis	Method	Reference	Result
**Appearance**	Occular	SP.FV-	
**Smell**	O.L	SP.FV-	
**Total ash**	Ph.Eur.Curr.Ed		5%
**Ash, insoluble in HCl**	Ph.Eur.Curr.Ed		1%
**Loss on drying**	Ph.Eur.Curr.Ed		3%
**Identity (TLC)**	SM-010	= ref	= ref
**HPLC**			
Eleuth B	SM-066		0,084 mg/dkl
Eleuth E	SM-066		0,119 mg/dkl
Σ Eleutherosides			0,203 mg/dkl
Andrographolide			8,15 mg/dkl
DDD-andr			1,50 mg/dkl
Σ Andr + DDD-andr			9,65 mg/dkl
Neoandr			3,44 mg/dkl
Weight including capsule	Ph.Eur.Curr.Ed	440 ± 33 mg	418 ± 4 mg
Weight without capsule	-	364 ± 27 mg	341 ± 4 mg
Diameter	-		6,9 ± 0,1 mm
Length	-		19,3 ± 0,1 mm
Disintegration	Ph.Eur.Curr.Ed (SM-032)	≤ 30 min	<30 min
		**Reference**	**Released**
**Microbial contamination**	Ph.Eur.Curr.Ed	Ph.Eur.Cat B	conform

Date of analysis: 111101

Analyst, sign: [illegible]

Date of release: 20111114

QP-responsible: [illegible]

**Comments:**	Manufacturing date:<br><br>Expiry date (two years):

the nature network®

PhytoLab

PhytoLab GmbH & Co.KG
Dutendorfer Straße 5-7
91487 Vestenbergsgreuth
Germany
Contact at PhytoLab:
Annette Reichel
Tel: +49 9163 88-188
Fax: +49 9163 88-379
annette.reichel@phytolab.de

Date: 9.11.11

Cust.No: 91778

SAL-akkreditiertes Prüflaboratorium Reg.Nr.: SAL- BY - G 104-03-08

PhytoLab GmbH & Co.KG Dutendorfer Straße 5-7 91487 Vestenbergsgreuth

Swedish Herbal Institute
Eva Vencel
Kövlingevägen 21
312 50 Vallberga
Sweden

Certificate of analysis

Report-No.:	13292590- 40001
Cust.-lot:	50145
Sample labelling:	Kan Jang capsule
Sample description:	135 capsules
Packing:	9x blister
Receipt of sample:	3.11.11

Test	Unit	Limit	Testresult
Microbiology: Category B, Herbal medicinal products containing, for example, extracts and/ or herbal drugs, with or without excipients, where the method of processing (for example, extraction) or, where appropriate, in the case of herbal drugs, of pre-treatment reduces the levels of organisms to below those stated for this category, according to Ph. Eur. 7.0, 5.1.8, SOP 803911:			Conform
Total aerobic microbial count (TAMC), Ph. Eur. 7.0, 2.6.12, SOP 300007	CFU/g	< = 50,000	50
Moulds (quantitative), Ph. Eur. 7.0, 2.6.12, SOP 300014	CFU/g		< 10
Yeasts (quantitative), Ph. Eur. 7.0, 2.6.12, SOP 300023	CFU/g		10
Total combined yeasts and moulds count (TYMC), Ph. Eur. 7.0, 2.6.12, SOP 300033	CFU/g	< = 500	10
Bile-tolerant gram-negative bacteria (semiquantitative, PN method), Ph. Eur. 7.0, 2.6.31, SOP 300044	CFU/g	< = 100	< 10
Escherichia coli (absence test), Ph. Eur.7.0, 2.6.31, SOP 300058	/g	Absent	Absent
Salmonella (absence test), Ph. Eur. 7.0, 2.6.31, SOP 300122	/25 g	Absent	Absent

Information on the method employed and on method characteristics is available to customers on request. The results apply to the supplied sample. Copying of the document is only permissible without any modification of the document.

the nature network®

PhytoLab

Certificate of analysis

Report-No.: 13292590- 40001
Cust.-lot: 50145
Sample labelling: Kan Jang capsule

Test	Unit	Limit	Testresult

Vestenbergsgreuth, 9.11.11

C. Borst

Dr. Claudia Borst

2011.11.09/BV

Svenska Örtmedicinska Institutet
Swedish Herbal Institute

Kan Jang Andrographis capsules, blister
HPTLC
50 145

Method: SM-010

VIS

Ref 3400039 | Test 50145

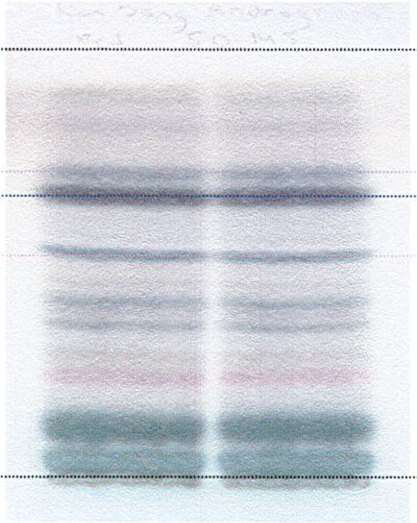

DDD-and
R_f: 0,71
Andr
R_f: 0,66
Neo-and
R_f: 0,52

254nm

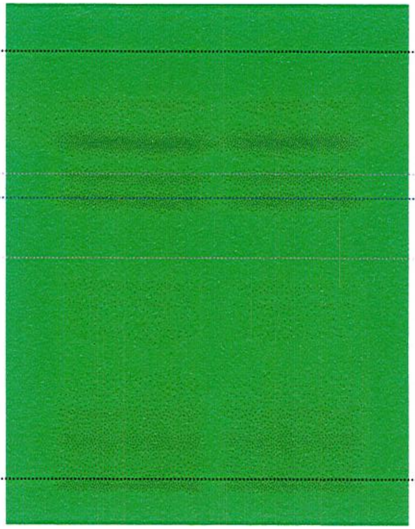

366nm

Front

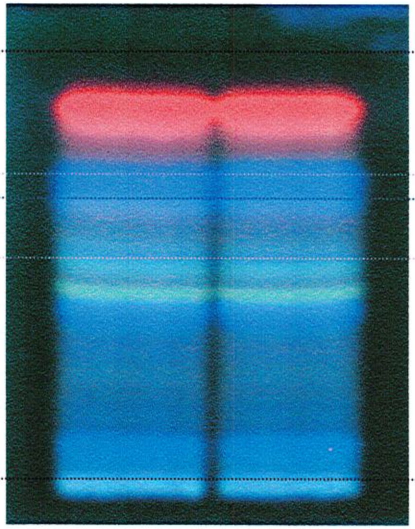

Start

Absorbent: Silica gel 60 F_{254} precoated HPTLC plates
Solvent system: Etylacetate : methanol : water (38 : 7 : 4)
Spray reagents: vanillin-sulphuric acid solution

Sign: Matilda

Date: 2011-11-03

PhytoLab GmbH & Co.KG
Dutendorfer Straße 5-7
91487 Vestenbergsgreuth
Germany
Contact at PhytoLab:
Annette Reichel
Tel: +49 9163 88-188
Fax: +49 9163 88-379
annette.reichel@phytolab.de

PhytoLab GmbH & Co.KG Dutendorfer Straße 5-7 91487 Vestenbergsgreuth

Swedish Herbal Institute
Eva Vencel
Kövlingevägen 21
312 50 Vallberga
Sweden

Date: 9.11.11

Cust.No: 91778

SAL-akkreditiertes Prüflaboratorium Reg.Nr.: SAL-BY-G 104-03-09

Certificate of analysis

Report-No.:	13292590- 40001
Cust.-lot:	50145
Sample labelling:	Kan Jang capsule
Sample description:	135 capsules
Packing:	9x blister
Receipt of sample:	3.11.11

Test	Unit	Limit	Testresult
Microbiology: Category B, Herbal medicinal products containing, for example, extracts and/ or herbal drugs, with or without excipients, where the method of processing (for example, extraction) or, where appropriate, in the case of herbal drugs, of pre-treatment reduces the levels of organisms to below those stated for this category, according to Ph. Eur. 7.0, 5.1.8, SOP 803911:			Conform
Total aerobic microbial count (TAMC), Ph. Eur. 7.0, 2.6.12, SOP 300007	CFU/g	< = 50,000	50
Moulds (quantitative), Ph. Eur. 7.0, 2.6.12, SOP 300014	CFU/g		< 10
Yeasts (quantitative), Ph. Eur. 7.0, 2.6.12, SOP 300023	CFU/g		10
Total combined yeasts and moulds count (TYMC), Ph. Eur. 7.0, 2.6.12, SOP 300033	CFU/g	< = 500	10
Bile-tolerant gram-negative bacteria (semiquantitative, PN method), Ph. Eur. 7.0, 2.6.31, SOP 300044	CFU/g	< = 100	< 10
Escherichia coli (absence test), Ph. Eur.7.0, 2.6.31, SOP 300058	/g	Absent	Absent
Salmonella (absence test), Ph. Eur. 7.0, 2.6.31, SOP 300122	/25 g	Absent	Absent

Information on the method employed and on method characteristics is available to customers on request. The results apply to the supplied sample. Copying of the document is only permissible without any modification of the document.

Certificate of analysis

Report-No.: 13292590- 40001
Cust.-lot: 50145
Sample labelling: Kan Jang capsule

Test	Unit	Limit	Testresult

Vestenbergsgreuth, 9.11.11

C. Borst

Dr. Claudia Borst

2011.11.15 /[illegible]

Svenska Örtmedicinska Institutet
Swedish Herbal Institute

Certificate of analysis

Product: Andrographis pan. extr. siccum **Batch no:** 1521114 Internal spec no

Batch no., previous extract: 1421047 Batch no., drug 1010247 SP EX-001

Parameter of analysis	Method	Reference	Result
Appearence	Occular	SP.EX-001	= ref
Loss on drying	Ph.Eur.Curr.Ed	≤ 5%	2 %
pH in 5% H2O-solution	Ph.Eur.Curr.Ed	5 - 7	6
Identity TLC	SM-010	= ref	= ref
Andrographolide	SM-118	- %	3,38 %
14-deoxy-11,12-didehydroandrographolide	SM-118	- %	0,64 %
Σ Andrographolides	SM-118	4.2-8.4%	4,02 % *
Σ Andrographolides in native extract	SM-118	6.0-14.0%	6,93 %
Neoandrographolide	SM-118	n.a.	1,49 %
Methylparahydroxybenzoate	SM-121	0.2-0.6%	0,35 %
Nativ ratio	SM-087	4.5-8.0 : 1	6,7 : 1
Microbiology	Ph.Eur Curr.Ed. 5.1.8	Ph. Eur. Cat B	conform
Ethanol	Ph.Eur.Curr.Ed. 2.4.24	≤2%	0,9 %

Date of analysis 2011.10.21 **Date of release** 20111031

Analyst signature [signature] **QP-responsible** [signature]

**Comments:** Ratio: 3,9:1<br>* Andrographolides are out of limits, but in the nativ extract are in the limits.	**Manufacturing date**<br>2011.10.18<br>**Expiry date (three years)**<br>2014-11

Empower 2 software

Sample Name:
Andr 1521114

Rapport_extrakt

Method: SM-121		Acquired By:	EvaV
Sample Type:	Unknown	Date Acquired:	2013-03-08 13:54:49 CET,
Vial:	1:A,1	Acq. Method Set:	Kons med andr
Injection #:	1, 2, 3	Date Processed:	2013-03-08 14:09:51 CET,
Injection Volume:	2.00 ul	Processing Method	Kons med andr
Run Time:	4.5 Minutes	Channel Name:	256 nm
Sample Set Name:	Andr 114 047	Column no.:	01363035115404

Component Results
SampleName: Andr 1521114

	SampleName	Vial	Inj	SampleWeight	Dilution	RT	Area	Height	Name	Percent_amount
1	Andr 1521114	1:A,1	3	7.61000	1.00000	2.867	653670	191651	Methyl-4-hydroxybenzoate	0.3491
2	Andr 1521114	1:A,1	1	7.61000	1.00000	2.868	656709	191503	Methyl-4-hydroxybenzoate	0.3507
3	Andr 1521114	1:A,1	2	7.61000	1.00000	2.873	654695	191117	Methyl-4-hydroxybenzoate	0.3496
Mean						2.87	655025			0.350
Std. Dev.						0.00	1546			0.001
% RSD						0.1	0.2			0.2

Result	***%***	***Calculated***	***% Found***
Methyl-4-hydroxybenzoate:	0,35 %	0,365 %	96 %

Chromatogram

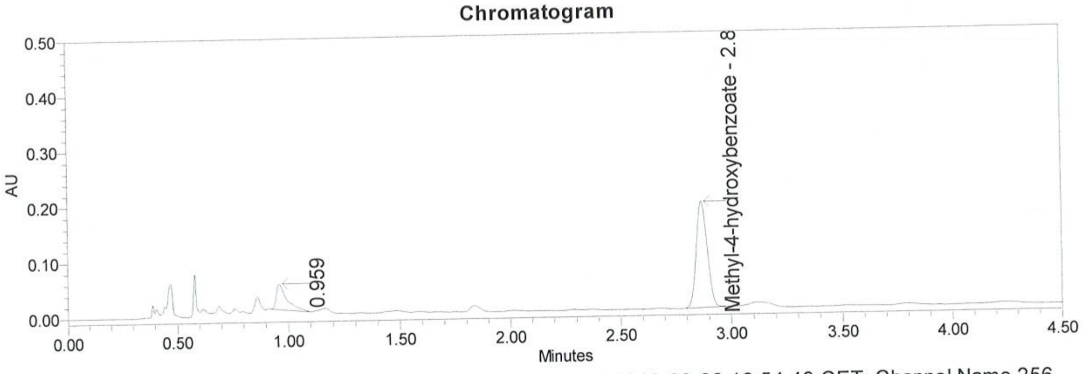

SampleName Andr 1521114; Vial 1:A,1; Date Acquired 2013-03-08 13:54:49 CET; Channel Name 256 nm

Sign.: W

Svenska Örtmedicinska Institutet
Swedish Herbal Institute

Andrographis siccum
HPTLC
15 21114

Method: SM-010

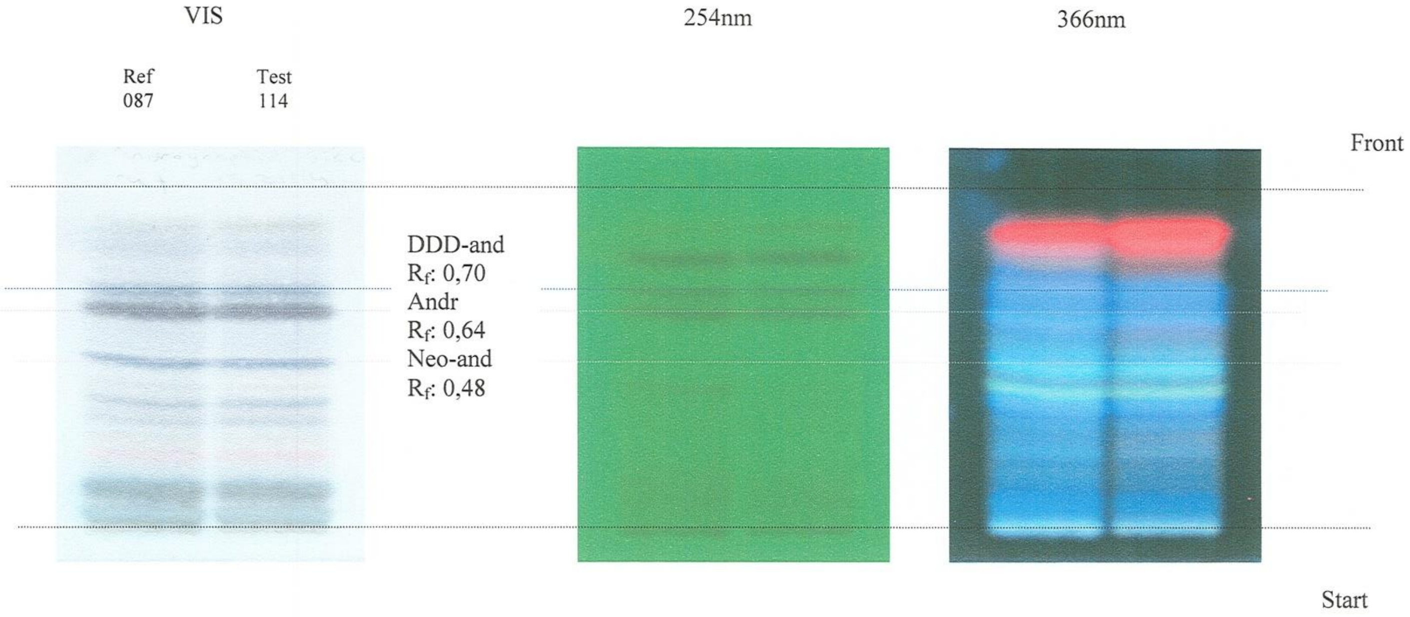

Absorbent:	Silica gel 60 F_{254} precoated HPTLC plates
Solvent system:	Etylacetate : methanol : water (38 : 7 : 4)
Spray reagents:	vanillin-sulphuric acid solution

Sign: Matilda

Date: 2011-10-21

Empower 2 software

Processing Method Andrographis

Start Date 2011-10-24 10:55:58 CEST

SampleName Andr 1521114

Name Andrographolide, Deoxy-didehydroandrographolide, Neoandrographolide

Current Date 2011-10-24
Software Empower 2 Software Build 2154
Column no.: Acquity UPLC BEH C18 1.7µm 01773000715504
Channel 205 nm, 223 nm, 254 nm
Run Time 5.00 Minutes

Component Results
Name: Andrographolide

	SampleName	Vial	Injection	SampleWeight	RT	Area	Height	Name	Percent_amount
1	Andr 1521114	1:A,4	3	2.10560	1.968	479849	298198	Andrographolide	3.381
2	Andr 1521114	1:A,4	1	2.10560	1.970	479741	298176	Andrographolide	3.381
3	Andr 1521114	1:A,4	2	2.10560	1.971	480825	299764	Andrographolide	3.388
Mean									3.383
Std. Dev.									0.004
% RSD									0.12

Component Results
Name: Neoandrographolide

	SampleName	Vial	Injection	SampleWeight	RT	Area	Height	Name	Percent_amount
1	Andr 1521114	1:A,4	3	2.10560	2.919	177917	159208	Neoandrographolide	1.487
2	Andr 1521114	1:A,4	2	2.10560	2.919	178163	159333	Neoandrographolide	1.489
3	Andr 1521114	1:A,4	1	2.10560	2.920	177943	159193	Neoandrographolide	1.487
Mean									1.487
Std. Dev.									0.001
% RSD									0.08

Component Results
Name: Deoxy-didehydroandrographolide

	SampleName	Vial	Injection	SampleWeight	RT	Area	Height	Name	Percent_amount
1	Andr 1521114	1:A,4	3	2.10560	3.186	72694	52211	Deoxy-didehydroandrographolide	0.641
2	Andr 1521114	1:A,4	2	2.10560	3.187	72912	52385	Deoxy-didehydroandrographolide	0.643
3	Andr 1521114	1:A,4	1	2.10560	3.188	72656	52187	Deoxy-didehydroandrographolide	0.641
Mean									0.642
Std. Dev.									0.001
% RSD									0.19

**Results**	**%**
**Andrographolide:**	3,38 %
**Neoandrographolide:**	1,49 %
**Deoxy-11,12-didehydroandrographolide:**	0,64 %
**Total :**	5,51 %

Sign.: [illegible]

Chromatogram

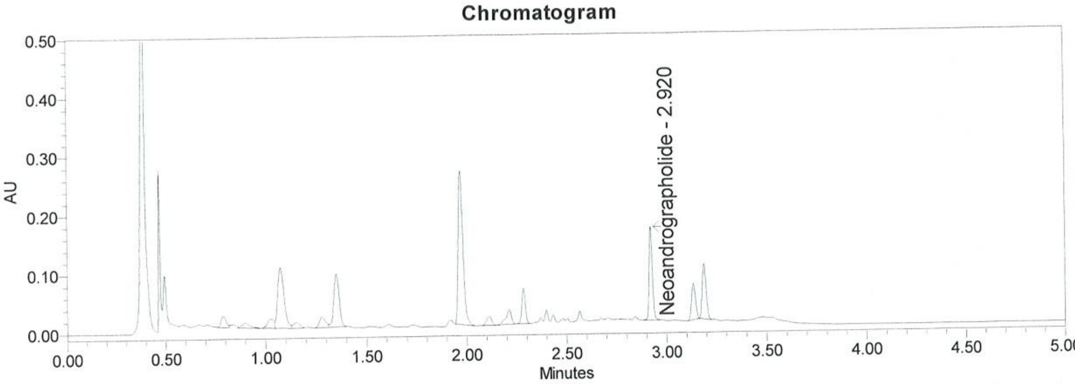

SampleName Andr 1521114; Vial 1:A,4; Injection 1; Date Acquired 2011-10-24 11:49:48 CEST; Channel Name 205 nm

Chromatogram

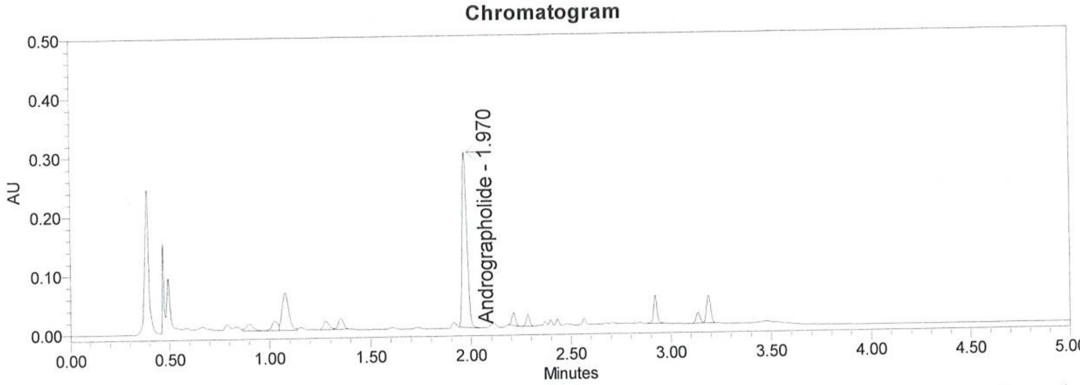

SampleName Andr 1521114; Vial 1:A,4; Injection 1; Date Acquired 2011-10-24 11:49:48 CEST; Channel Name 223 nm

Chromatogram

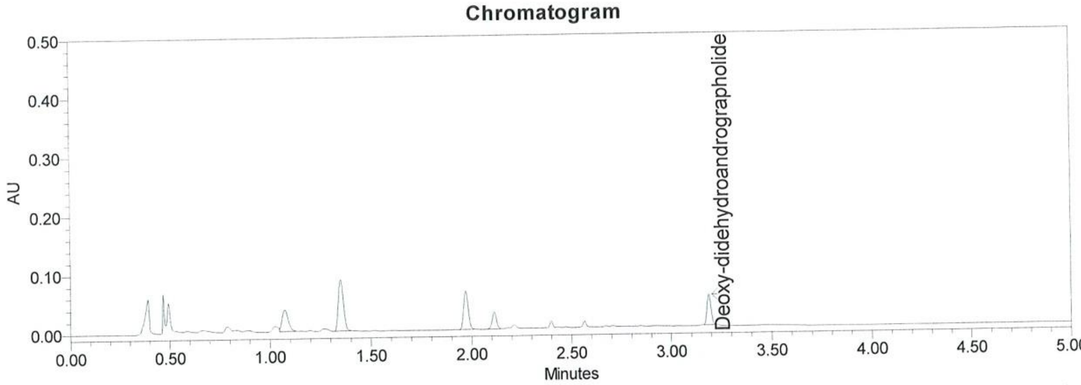

SampleName Andr 1521114; Vial 1:A,4; Injection 1; Date Acquired 2011-10-24 11:49:48 CEST; Channel Name 254 nm

Sign.:

the nature network®

PhytoLab

PhytoLab GmbH & Co.KG Dutendorfer Straße 5-7 91487 Vestenbergsgreuth

Swedish Herbal Institute
Eva Vencel
Kövlingevägen 21
312 50 Vallberga
Sweden

PhytoLab GmbH & Co.KG
Dutendorfer Straße 5-7
91487 Vestenbergsgreuth
Germany
Contact at PhytoLab:
Annette Reichel
Tel: +49 9163 88-188
Fax: +49 9163 88-379
annette.reichel@phytolab.de

Date: 31.10.11

Cust.No: 91778

SAL-akkreditiertes Prüflaboratorium
Reg.Nr.: SAL-BY-G 104.03.09

Certificate of analysis

Report-No.: 13248486- 40001
Cust.-lot: 15 21114
Sample labelling: Andrographis siccum
Sample description: green, fine powder
Packing: pe container with screw closure
Receipt of sample: 21.10.11

Test	Unit	Limit	Testresult
Microbiology: Category B, Herbal medicinal products containing, for example, extracts and/ or herbal drugs, with or without excipients, where the method of processing (for example, extraction) or, where appropriate, in the case of herbal drugs, of pre-treatment reduces the levels of organisms to below those stated for this category, according to Ph. Eur. 7.0, 5.1.8, SOP 803911:			Conform
Total aerobic microbial count (TAMC), Ph. Eur. 7.0, 2.6.12, SOP 300007	CFU/g	< = 50,000	50
Moulds (quantitative), Ph. Eur. 7.0, 2.6.12, SOP 300014	CFU/g		< 10
Yeasts (quantitative), Ph. Eur. 7.0, 2.6.12, SOP 300023	CFU/g		< 10
Total combined yeasts and moulds count (TYMC), Ph. Eur. 7.0, 2.6.12, SOP 300033	CFU/g	< = 500	< 10
Bile-tolerant gram-negative bacteria (semiquantitative, PN method), Ph. Eur. 7.0, 2.6.31, SOP 300044	CFU/g	< = 100	< 10
Escherichia coli (absence test), Ph. Eur.7.0, 2.6.31, SOP 300058	/g	Absent	Absent
Salmonella (absence test), Ph. Eur. 7.0, 2.6.31, SOP 300122	/25 g	Absent	Absent

Information on the method employed and on method characteristics is available to customers on request. The results apply to the supplied sample. Copying of the document is only permissible without any modification of the document.

the nature network®

PhytoLab

Certificate of analysis

Report-No.: 13248486- 40001
Cust.-lot: 15 21114
Sample labelling: Andrographis siccum

Test	Unit	Limit	Testresult

Vestenbergsgreuth, 31.10.11

C Borst

Dr. Claudia Borst

2011.10.31 / [illegible]

the nature network®

PhytoLab

PhytoLab GmbH & Co.KG Dutendorfer Straße 5-7 91487 Vestenbergsgreuth

Swedish Herbal Institute
Eva Vencel
Kövlingevägen 21
312 50 Vallberga
Sweden

PhytoLab GmbH & Co.KG
Dutendorfer Straße 5-7
91487 Vestenbergsgreuth
Germany
Contact at PhytoLab:
Annette Reichel
Tel: +49 9163 88-188
Fax: +49 9163 88-379
annette.reichel@phytolab.de

Date: 31.10.11

Cust.No: 91778

SAL-akkreditiertes Prüflaboratorium Reg.Nr.: SAL- BY - G 104-03-09

Certificate of analysis

Report-No.:	13248486- 40001
Cust.-lot:	15 21114
Sample labelling:	Andrographis siccum
Sample description:	green, fine powder
Packing:	pe container with screw closure
Receipt of sample:	21.10.11

Test	Unit	Limit	Testresult
Microbiology: Category B, Herbal medicinal products containing, for example, extracts and/ or herbal drugs, with or without excipients, where the method of processing (for example, extraction) or, where appropriate, in the case of herbal drugs, of pre-treatment reduces the levels of organisms to below those stated for this category, according to Ph. Eur. 7.0, 5.1.8, SOP 803911:			Conform
Total aerobic microbial count (TAMC), Ph. Eur. 7.0, 2.6.12, SOP 300007	CFU/g	< = 50,000	50
Moulds (quantitative), Ph. Eur. 7.0, 2.6.12, SOP 300014	CFU/g		< 10
Yeasts (quantitative), Ph. Eur. 7.0, 2.6.12, SOP 300023	CFU/g		< 10
Total combined yeasts and moulds count (TYMC), Ph. Eur. 7.0, 2.6.12, SOP 300033	CFU/g	< = 500	< 10
Bile-tolerant gram-negative bacteria (semiquantitative, PN method), Ph. Eur. 7.0, 2.6.31, SOP 300044	CFU/g	< = 100	< 10
Escherichia coli (absence test), Ph. Eur.7.0, 2.6.31, SOP 300058	/g	Absent	Absent
Salmonella (absence test), Ph. Eur. 7.0, 2.6.31, SOP 300122	/25 g	Absent	Absent

Information on the method employed and on method characteristics is available to customers on request. The results apply to the supplied sample. Copying of the document is only permissible without any modification of the document.

the nature network®

PhytoLab

Certificate of analysis

Report-No.: 13248486- 40001
Cust.-lot: 15 21114
Sample labelling: Andrographis siccum

Test	Unit	Limit	Testresult

Vestenbergsgreuth, 31.10.11

C Borst

Dr. Claudia Borst

2011.11.14/ [illegible]

eurofins

Svenska Örtmedicinska Institutet
Eva Vencel
Kovlingevägen 21
SE-312 50 Vallberga

Eurofins Pharma A/S
Strandesplanaden 110
DK-2665 Vallensbæk Strand
Denmark

Tel. +45 70 22 42 66
Fax +45 70 22 42 55
eurofins@eurofins.dk
www.eurofins.dk

Date
02 November 2011
Our ref.
942687/SLH

Analytical report

Sample received	Test sample
20 October 2011	Andrograhis extract siccum

Customer's journal no/ project title:

-

Label(s): Batch no. 15 21114

Information from client: -

Methods: Specific references are listed with the test results

Reference: -

Results: Results are reported on page 2-3.

Comments: Samples are analysed by the laboratory:

Dr. Fintelmann und Dr. Meyer, GMBH, Germany

Signatures

Sandy Leth
MSc (Pharm)

eurofins | **Fintelmann und Meyer GMP GmbH**

Eurofins Fintelmann und Meyer GMP GmbH
Großmoorbogen 25
D-21079 Hamburg
GERMANY

Tel: +49 40 49 294 670
Fax: +49 40 49 294 699

info@fintelmann-meyer.de
www.fintelmann-meyer.de

Eurofins Fintelmann und Meyer GMP GmbH · Großmoorbogen 25 · D-21079 Hamburg

Svenska Örtmedicinska Institutet
attn. Mrs. Eva Vencel
Kövlingevägen 21
31250
SCHWEDEN

Report date 01.11.2011

Analytical report AR-11-FG-009993-01

Sample Code 714-2011-00017541

**Reference**	Andrographis extrakt siccum<br>Batch nr.15 21114<br>19.10.2011
**Sample sender**	Mrs. Eva Vencel
**Prescriber**	Eurofins Pharma A/S
**Reception date time**	21.10.2011
**Client sample code**	9426-8701
**Packaging**	plastic container
**Reception temperature**	room temperature
**Storage conditions**	room temperature
**Start analysis**	21.10.2011
**End analysis**	01.11.2011

Test results

FGL15 Ethanol
Method EP 7.2, on the basis of method 2.4.24

Ethanol	0.9	%

JUDGEMENT

With regard to the analysed parameters the tested sample material meets the respective requirements of the customer.

"Analysis required by Eurofins Pharma A/S"

The results of examination refer exclusively to the checked samples.
Duplicates - even in parts - must be authorized by the test laboratory in written form.
Eurofins Fintelmann und Meyer GMP GmbH · Großmoorbogen 25 · D-21079 Hamburg
Business Unit Manager: Dr. Helge Neumann-Hensel · Dr. Markus Brandmeier Place of performance shall be Hamburg, Germany. Place of jurisdiction shall be Hamburg, Germany.
Our General Terms & Conditions of Sales are applicable.
VAT No.: DE 269767648
Norddeutsche Landesbank Hannover · Kto. Nr. 151 358 628 · BLZ 250 500 00 IBAN: DE90250500000151358625
BIC: NOLADE2HXXX

Our General Terms & Conditions of Sales are applicable.

eurofins

Fintelmann und Meyer GMP GmbH

Analytical report AR-11-FG-009993-01
Sample Code 714-2011-00017541

Signature

Dr. H. Neumann-Hensel / B. Kohnke / K. Oelkers / Dr. T. Rathjen
(General manager) (Department management)

eurofins
Eurofins Pharma A/S
Strandesplanaden [illegible]
2665 Vallensbæk [illegible]
Tlf. 70 22 4[illegible]
02. NOV. 2011

The results of examination refer exclusively to the checked samples.
Duplicates - even in parts - must be authorized by the test laboratory in written form.
Eurofins Fintelmann und Meyer GMP GmbH · Großmoorbogen 25 · D-21079 Hamburg
Business Unit Manager: Dr. Helge Neumann-Hensel • Dr. Markus Brandmeier Place of performance shall be Hamburg,
Germany. Place of jurisdiction shall be Hamburg, Germany
Our General Terms & Conditions of Sales are applicable.
VAT No.: DE 269757648
Norddeutsche Landesbank Hannover • Kto. Nr. 151 355 625 • BLZ 250 500 00 · IBAN: DE90250500000151355625
BIC: NOLADE2HXXX

Our General Terms & Conditions of Sales are applicable.

2011. 11.02 / SV

Svenska Örtmedicinska Institutet
Swedish Herbal Institute

Certificate of analysis

Doc. no. ANJE.003
Internal spec. no. SP.EX-003E

Product: **Eleutherococcus sent. extr. siccum**

Batch no: 1521077

Batch no., previous extract: 1421061

Batch no., drug: 1010237

Parameter of analysis	Method	Reference	Result
**Appearence**	Occular	= ref	= ref
**Loss on drying**	Ph Eur Curr Ed	< 5%	1,0%
**pH in 5% H2O-solution**	Ph Eur Curr Ed	4,5 - 6	5,03
**Identity (TLC)**	SM-018	= ref	= ref
**Identity of markers**	SM-094	=standards	= ref
**HPLC**			
Eleutherosid B + E in native	SM-073	1,2-2,9%	1,78 %
Eleutherosid B + E in total extr.			0,89 %
Potassium sorbate	SM-046	0,05-0,25%	0,10%
Mettylparahydroxybensoat	SM-046	0,05-0,25%	0,09%
		**Reference**	**Result**
**Ratio**			9:1
**Nativ ratio**		17 - 30:1	17:1
		**Reference**	**Result**
**Microbiology**	PhEur Curr Ed	Ph. Eur. Cat 3B (corrected) 11.03.07	Conform
**Ethanol**	PhEur Curr Ed	<1%	<1%

Date of analysis: 11.02.16

Date of release: 2011 -03- 08

Analyst signature: [signature]

QP-responsible: [signature]

**Comments:**	**Manufacturing date** 2011.02.17<br>**Expiry date (two years)** 2013-03

Checked by: (QC)	Eva Vencel [signature]	Date: 081021	Issue no: 11
Authorised by (QA):	Charlotte Nilsson CN	Date: 081021	Supersedes no: 10
Authorised by (QP):	Dan Magnusson [signature]	Date: 081021	

Svenska Örtmedicinska Institutet

Swedish Herbal Institute

Eleutherococcus siccum

TLC

15 21077

Metod: SM-018

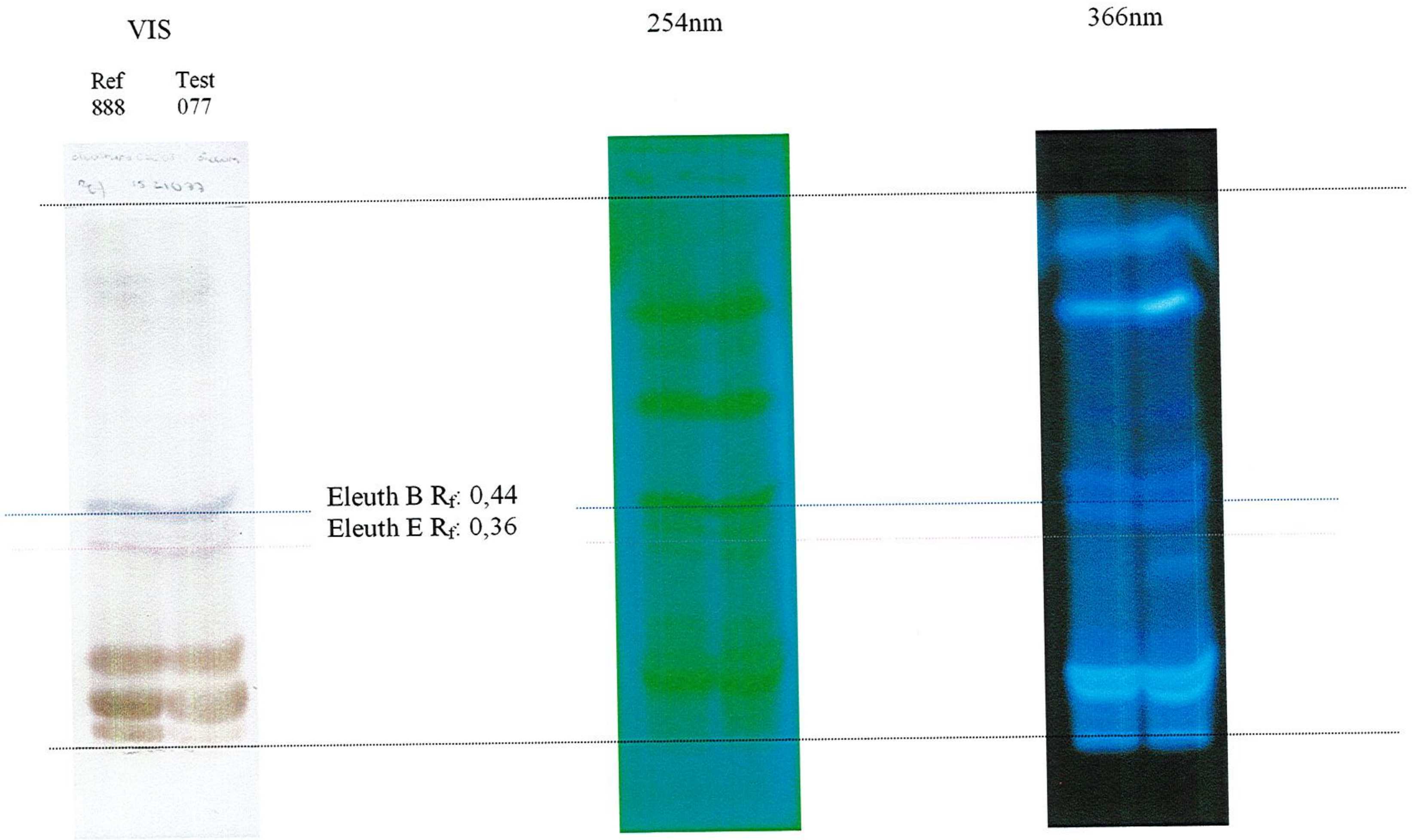

Absorbent: Silica gel 60 F_{254} precoated TLC plates
Solvent system: chloroform : methanol : water (70 : 40 : 10)
Spray reagents: vanillin-phosphoric acid

Sign: Matilda

Date: 2011-02-21

Empower 2 software

SampleName Eleuth 1521077

Current Date 2011-02-18
Software Empower 2 Software Build 215[illegible]
Column no.: 812269
Run Time 45.00 Minutes
Channel 254 nm, 262 nm

Processing Method Konsmedel_extr

Start Date 2011-02-17 13:17:40 CET

Potassiumsorbate
Methyl-4-hydroxybenzoate

Component Results
Name: Potassiumsorbate

	SampleName	RT	Injection Volume (uL)	SampleWeight	Area	Height	Name	Percent_amount
1	Eleuth 1521077	28.640	10.00	380.50000	3492182	60841	Potassiumsorbate	0.097
2	Eleuth 1521077	28.673	10.00	380.50000	3511265	61224	Potassiumsorbate	0.097
3	Eleuth 1521077	28.713	10.00	380.50000	3511119	60950	Potassiumsorbate	0.097
Mean					3504855			0.097
Std. Dev.					10975			0.000
% RSD					0.3			0.29

Component Results
Name: Methyl-4-hydroxybenzoate

	SampleName	RT	Injection Volume (uL)	SampleWeight	Area	Height	Name	Percent_amount
1	Eleuth 1521077	36.214	10.00	380.50000	1935028	27296	Methyl-4-hydroxybenzoate	0.095
2	Eleuth 1521077	36.269	10.00	380.50000	1849160	26945	Methyl-4-hydroxybenzoate	0.091
3	Eleuth 1521077	36.338	10.00	380.50000	1907624	27212	Methyl-4-hydroxybenzoate	0.094
Mean					1897271			0.093
Std. Dev.					43860			0.002
% RSD					2.3			2.18

Results	***%***	***Theoretical***	***Yield***
Potassiumsorbate:	0,097%	0,169%	57%
Methyl-4-hydroxybenzoate:	0,093%	0,169%	55%

Sign.: Mo

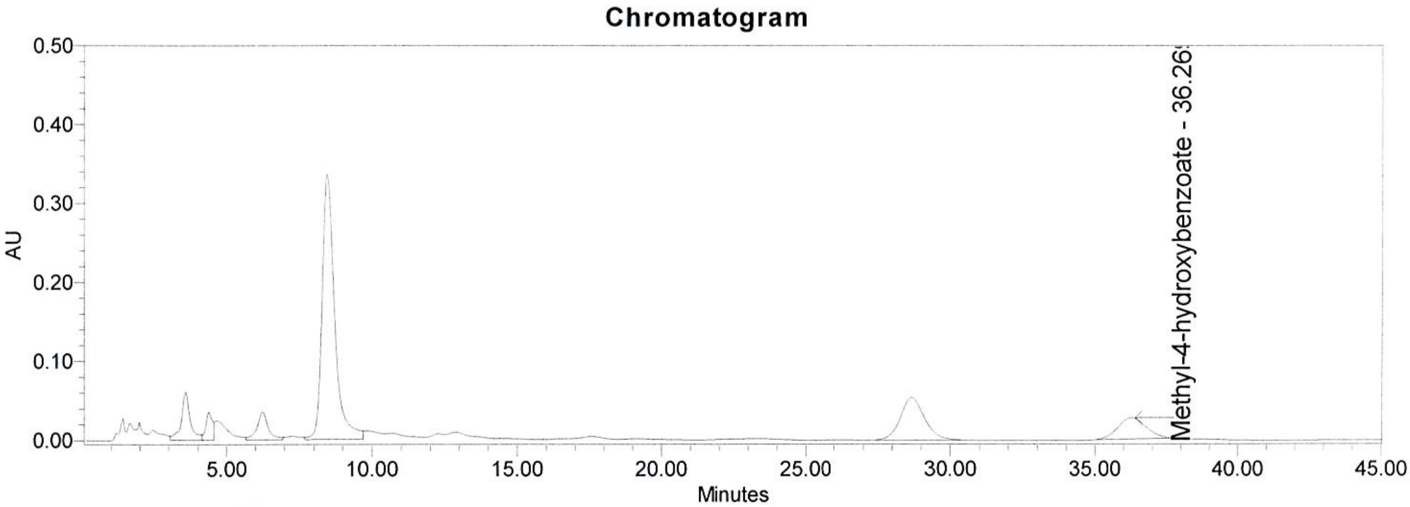

SampleName Eleuth 1521077; Vial 4; Date Acquired 2011-02-17 20:55:16 CET; Channel Name 254 nm

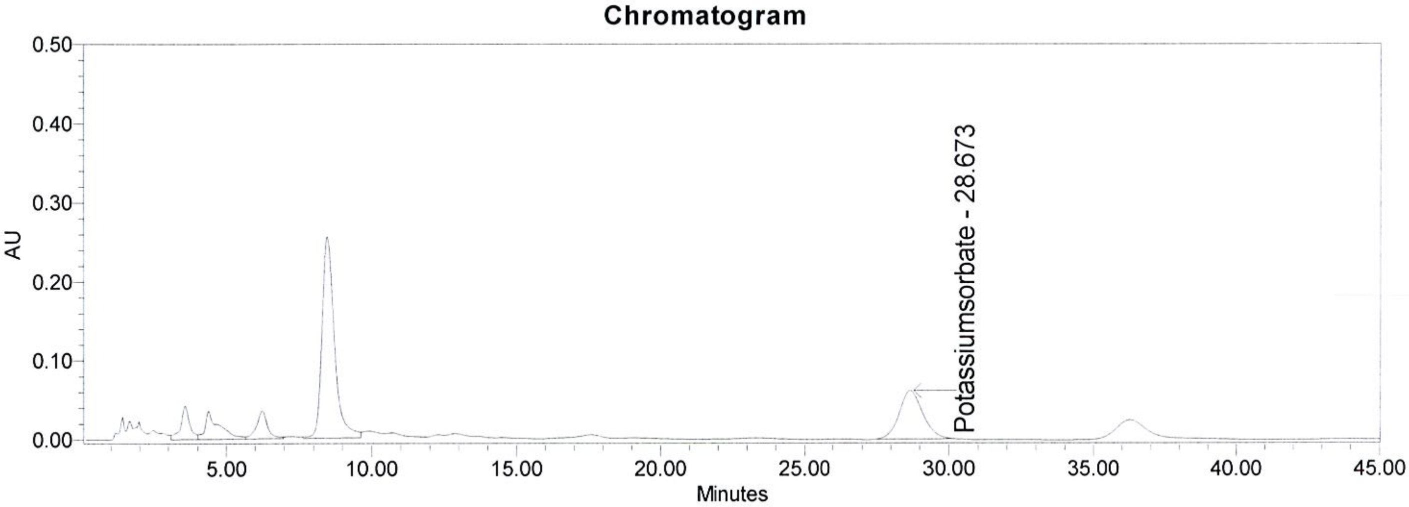

SampleName Eleuth 1521077; Vial 4; Date Acquired 2011-02-17 20:55:16 CET; Channel Name 262 nm

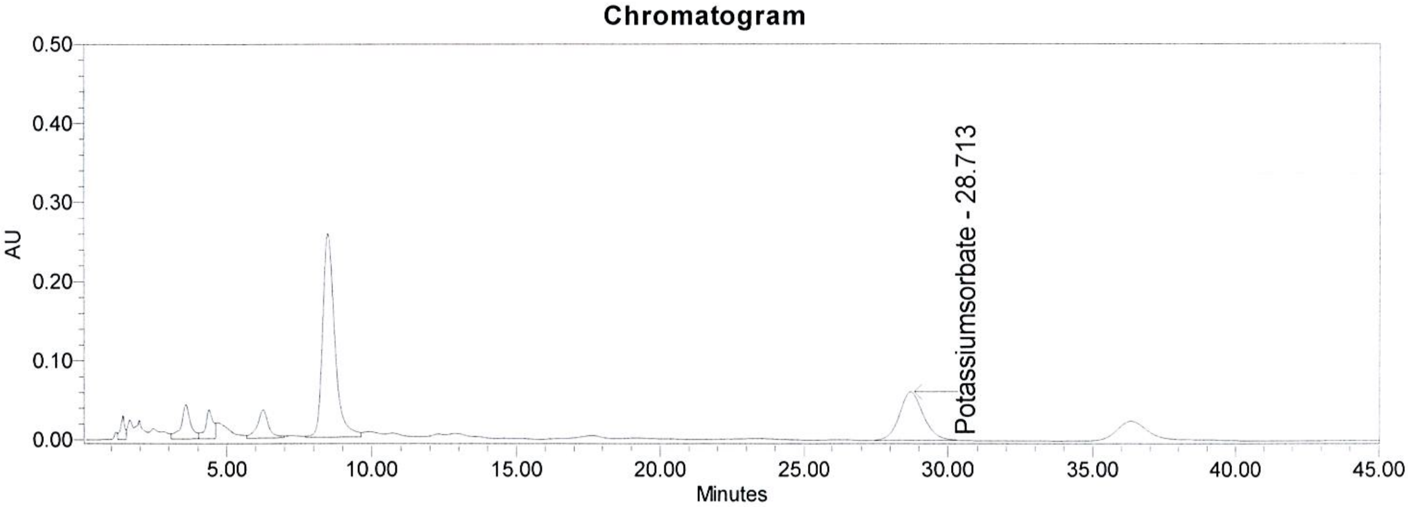

SampleName Eleuth 1521077; Vial 4; Date Acquired 2011-02-17 21:41:19 CET; Channel Name 262 nm

Sign.: Ma

Empower2 software

SampleName Eleuth 1521077

Rapport_extrakt

Software Empower 2 Software Build 215

Current Date 2011-02-17

Column no.: 937031

Processing Method Acax_validerad

Run Time 35.00 Minutes

Start Date 2011-02-16 13:34:43

Channel PDA_210.0nm, PDA_220.0nm

System Name HPLC

Eleutherosid B
Eleutherosid E

Name: Eleutherosid B

	SampleName	Injection Volume (ul)	SampleWeight	Dilution	RT	Area	Height	Name	Procent_Amount
1	Eleuth 1521077	20.00	83.20000	1.00000	5.746	1485129	84983	Eleutherosid B	0.384
2	Eleuth 1521077	20.00	83.20000	1.00000	5.797	1522082	84010	Eleutherosid B	0.394
3	Eleuth 1521077	20.00	83.20000	1.00000	5.808	1487197	84077	Eleutherosid B	0.384
Mean									0.387
Std. Dev.									0.006
% RSD									1.44

Name: Eleutherosid E

	SampleName	Injection Volume (ul)	SampleWeight	Dilution	RT	Area	Height	Name	Procent_Amount
1	Eleuth 1521077	20.00	83.20000	1.00000	18.193	2174703	127252	Eleutherosid E	0.507
2	Eleuth 1521077	20.00	83.20000	1.00000	18.215	2165848	129793	Eleutherosid E	0.504
3	Eleuth 1521077	20.00	83.20000	1.00000	18.236	2160819	131999	Eleutherosid E	0.503
Mean									0.505
Std. Dev.									0.002
% RSD									0.36

Results	*%*	*Theoretical*	*Yield*
Eleutherosid B:	0,39%	0,38	
Eleutherosid E:	0,50%	0,46	
Eleutherosides:	0,89%	0,84	106%

Chromatogram

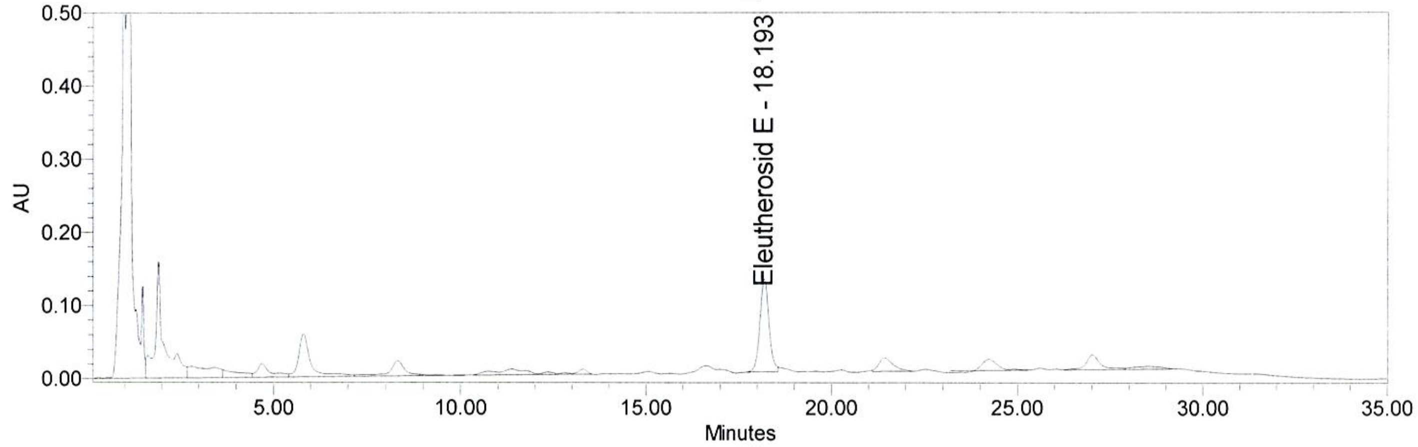

SampleName Eleuth 1521077; Vial 4; Date Acquired 2011-02-16 20:30:21 CET; Channel Name PDA_210.0nm

Sign.: [signature]

Chromatogram

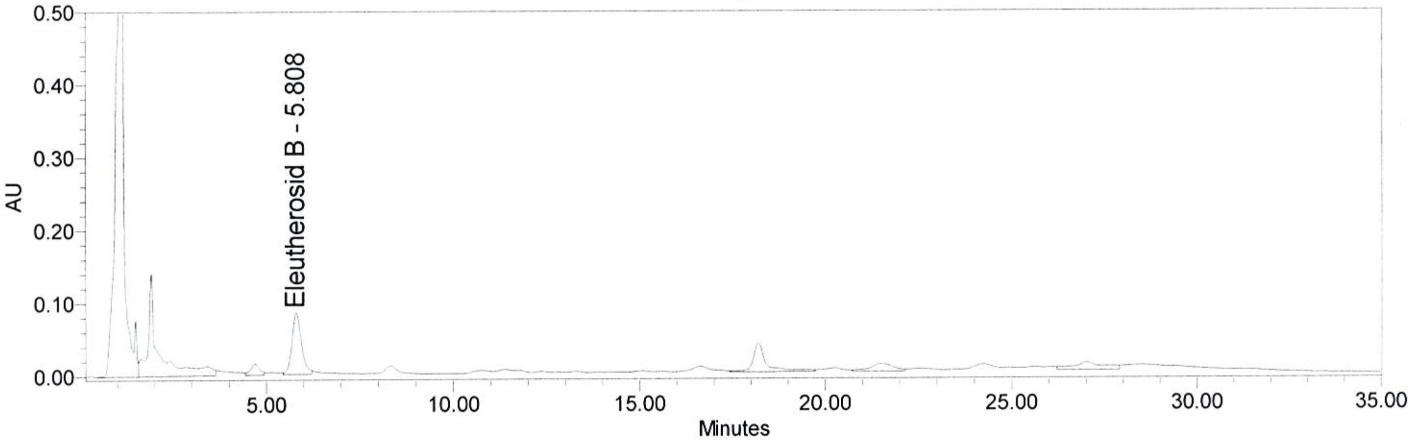

SampleName Eleuth 1521077; Vial 4; Date Acquired 2011-02-16 20:30:21 CET; Channel Name PDA_220.0nm

Chromatogram

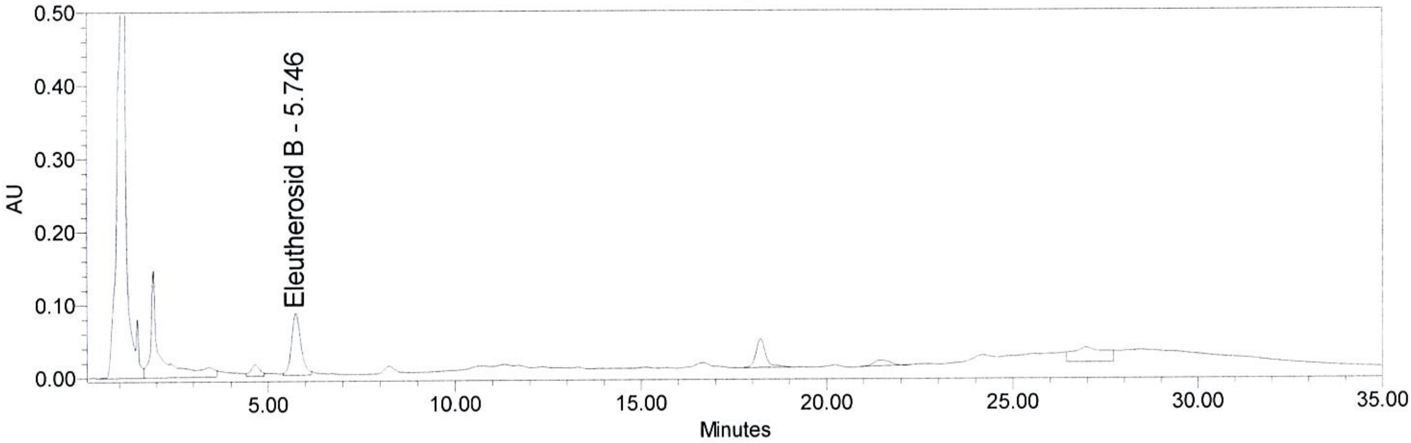

SampleName Eleuth 1521077; Vial 4; Date Acquired 2011-02-16 21:16:16 CET; Channel Name PDA_220.0nm

Chromatogram

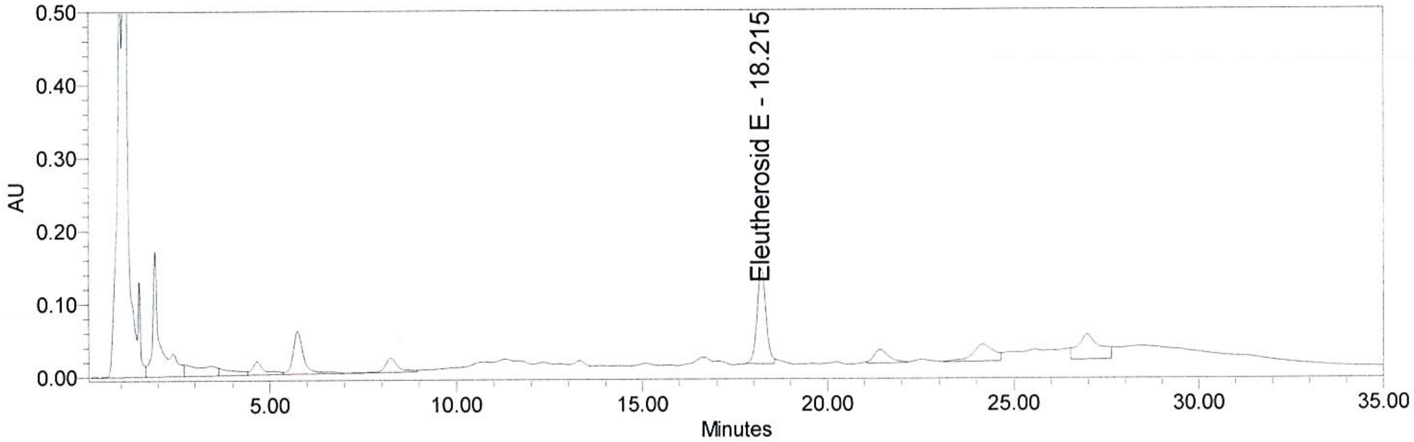

SampleName Eleuth 1521077; Vial 4; Date Acquired 2011-02-16 21:16:16 CET; Channel Name PDA_210.0nm

Sign.:

Empower 2 software

rocessing Method Acanth_PDA
un Time 35.00 Minutes
tart Date 2011-02-16 13:34:43 CET

Second identification with PDA

Eleutherosid B, Eleutherosid E

SampleName Eleuth 1521077

Current Date 2011-02-17
Software Empower 2 Software Build 2
Column no.: 937031
System Name HPLC

Spectrum Index Plot

Eleutherosid E - 18.193

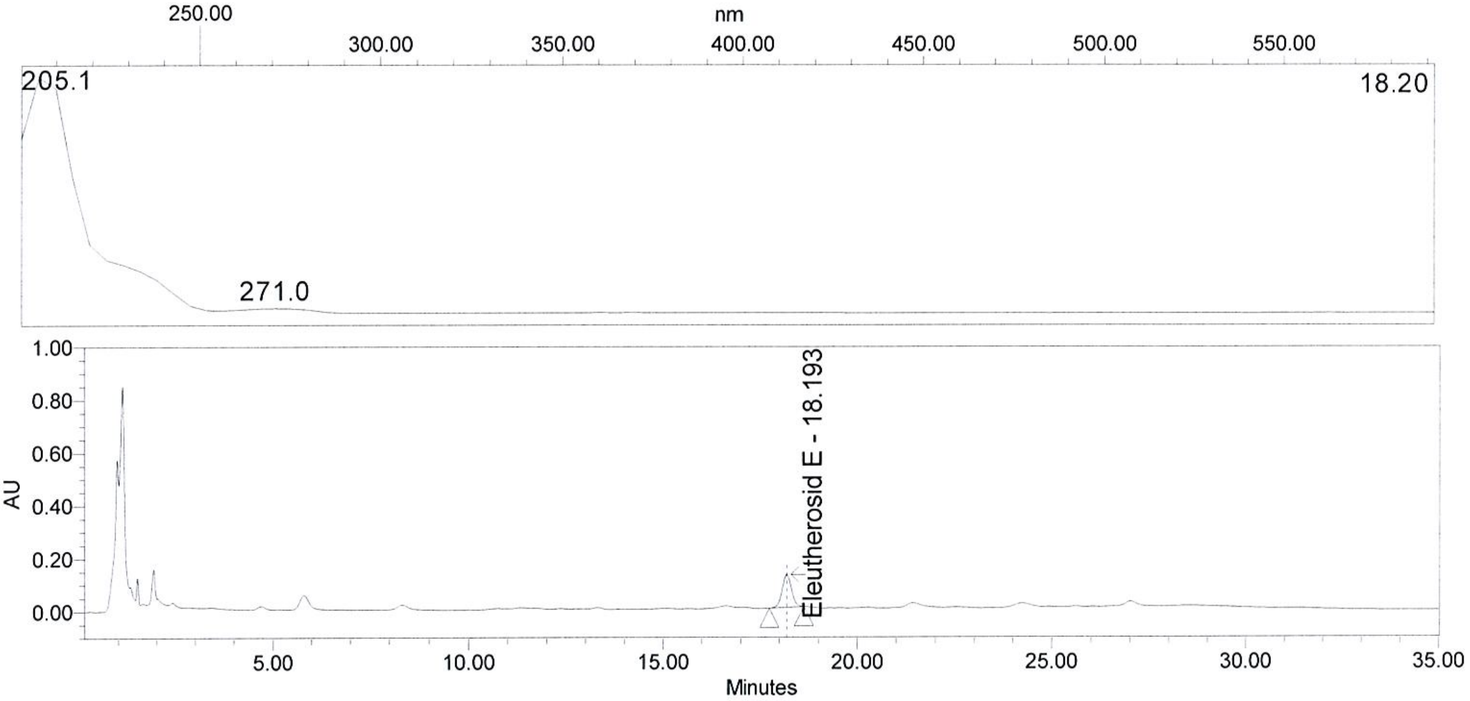

Spectrum Index Plot

Eleutherosid B - 5.808

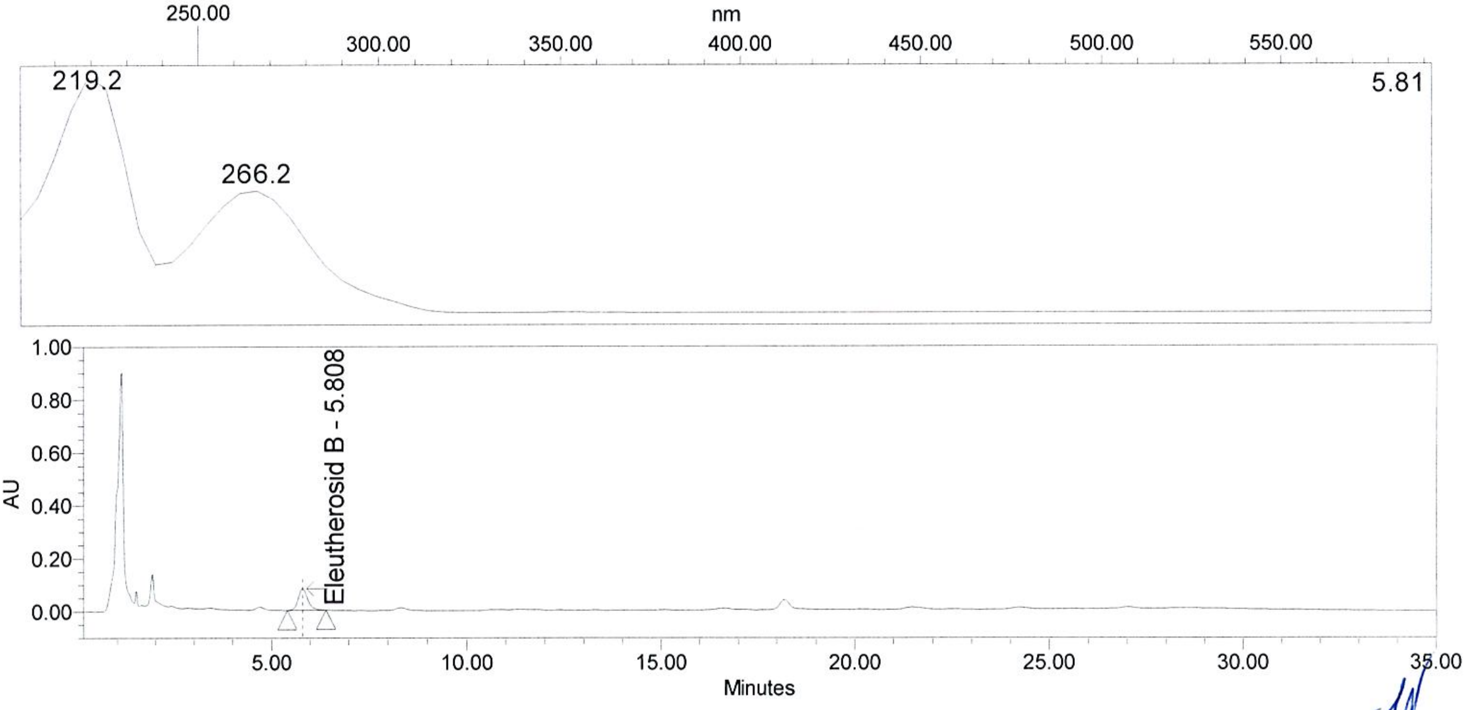

Sign.:

Match Plot

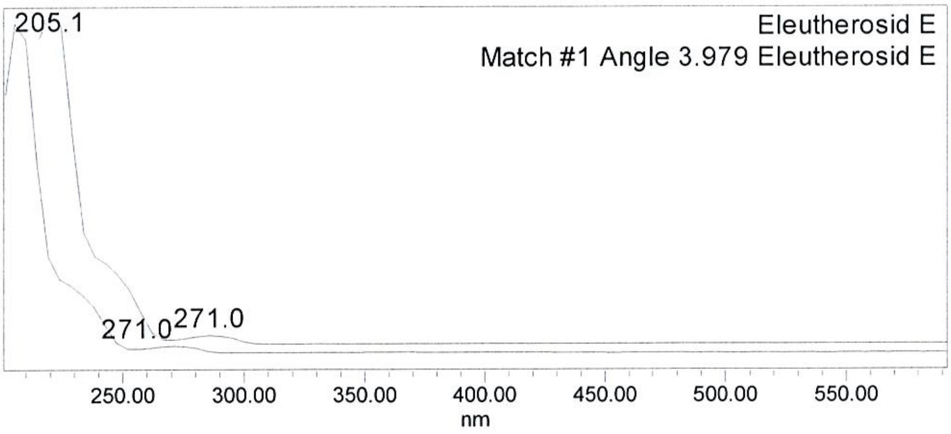

SampleName Eleuth 1521077 Vial 4 Injection 1 Date Acquired 2011-02-16 20:30:21 CET Name Eleutherosid E Match1 Angle 3.979 Match1 Threshold 1.204 Match1 Spect. Name Eleutherosid E

Match Plot

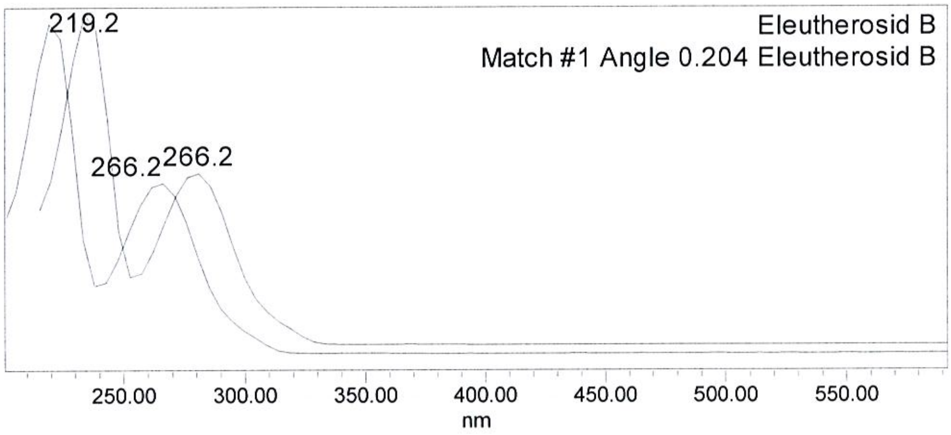

SampleName Eleuth 1521077 Vial 4 Injection 1 Date Acquired 2011-02-16 20:30:21 CET Name Eleutherosid B Match1 Angle 0.204 Match1 Threshold 1.120 Match1 Spect. Name Eleutherosid B

PDA Result Table

	Name	RT	Purity1 Angle	Purity1 Threshold	Match1 Spect. Name	Match1 Angle	Match1 Threshold
1	Eleutherosid B	5.808	0.301	1.236	Eleutherosid B	0.204	1.120
2	Eleutherosid E	18.193	1.293	1.382	Eleutherosid E	3.979	1.204

Sign.:

the nature network®

PhytoLab

PhytoLab GmbH & Co.KG
Dutendorfer Straße 5-7
91487 Vestenbergsgreuth
Germany
Contact at PhytoLab:
Annette Reichel
Tel: +49 9163 88-188
Fax: +49 9163 88-379
annette.reichel@phytolab.de

PhytoLab GmbH & Co.KG Dutendorfer Straße 5-7 91487 Vestenbergsgreuth

Swedish Herbal Institute
Eva Vencel
Kövlingevägen 21
312 50 Vallberga
Sweden

Date: 7.03.11

Cust.No: 91778

SAL-akkreditiertes Prüflaboratorium
Reg.Nr.: SAL-BY-G 104-03-09

Certificate of analysis

Report-No.: 12461844- 40001
Cust.-lot: 1521077
Sample labelling: Eleutherococcus sicc. extr
Sample description: brown powder
Packing: PE-container with screw closure
Receipt of sample: 24.02.11

Test	Unit	Limits	Testresult
Microbiology: Category B, Herbal medicinal products containing, for example, extracts and/ or herbal drugs, with or without excipients, where the method of processing (for example, extraction) or, where appropriate, in the case of herbal drugs, of pre-treatment reduces the levels of organisms to below those stated for this category, according to Ph. Eur. 6.7, 5.1.8, SOP 803911:			Conform
Total aerobic microbial count (TAMC), SOP 300007	CFU/g	< = 50,000	180
Moulds, SOP 300014	CFU/g		< 10
Yeasts, SOP 300023	CFU/g		30
Total combined yeasts and moulds count (TYMC), SOP 300033	CFU/g	< = 500	30
Bile-tolerant gram-negative bacteria (semiquantitative, PN method), SOP 300044	CFU/g	< = 100	< 10
Escherichia coli (absence test), SOP 300058	/g	Absent	Absent
Salmonella (qualitative), SOP 300122	/25 g	Absent	Absent

Information on the method employed and on method characteristics is available to customers on request. The results apply to the supplied sample. Copying of the document is only permissible without any modification of the document.

Vestenbergsgreuth, 7.03.11

Elke Lenzer

2011.03.14

the nature network®

PhytoLab

PhytoLab GmbH & Co.KG Dutendorfer Straße 5-7 91487 Vestenbergsgreuth

Swedish Herbal Institute
Eva Vencel
Kövlingevägen 21
312 50 Vallberga
Sweden

PhytoLab GmbH & Co.KG
Dutendorfer Straße 5-7
91487 Vestenbergsgreuth
Germany
Contact at PhytoLab:
Annette Reichel
Tel: +49 9163 88-188
Fax: +49 9163 88-379
annette.reichel@phytolab.de

Date: 7.03.11

Cust.No: 91778

SAL-akkreditiertes Prüflaboratorium Reg.Nr.: SAL-BY-G 104-03-09

Certificate of analysis

Report-No.: 12461844- 40001
Cust.-lot: 1521077
Sample labelling: Eleutherococcus sicc. extr
Sample description: brown powder
Packing: PE-container with screw closure
Receipt of sample: 24.02.11

Test	Unit	Limits	Testresult
Microbiology: Category B, Herbal medicinal products containing, for example, extracts and/ or herbal drugs, with or without excipients, where the method of processing (for example, extraction) or, where appropriate, in the case of herbal drugs, of pre-treatment reduces the levels of organisms to below those stated for this category, according to Ph. Eur. 6.7, 5.1.8, SOP 803911:			Conform
Total aerobic microbial count (TAMC), SOP 300007	CFU/g	<= 50,000	180
Moulds, SOP 300014	CFU/g		< 10
Yeasts, SOP 300023	CFU/g		30
Total combined yeasts and moulds count (TYMC), SOP 300033	CFU/g	<= 500	30
Bile-tolerant gram-negative bacteria (semiquantitative, PN method), SOP 300044	CFU/g	<= 100	< 10
Escherichia coli (absence test), SOP 300058	/g	Absent	Absent
Salmonella (qualitative), SOP 300122	/25 g	Absent	Absent

Information on the method employed and on method characteristics is available to customers on request. The results apply to the supplied sample. Copying of the document is only permissible without any modification of the document.

Vestenbergsgreuth, 7.03.11

Elke Lenzer

2011.03.07

Svenska Örtmedicinska Institutet
Eva Vencel
Kövlingevägen 21
SE-312 50 Vallberga

Eurofins Pharma A/S
Strandesplanaden 110
DK-2665 Vallensbæk Strand
Denmark

Tel. **+45 70 22 42 66**
Fax **+45 70 22 42 55**
eurofins@eurofins.dk
www.eurofins.dk

Date
01 March 2011
Our ref.
940848/SLH

Analytical report

**Sample received**	**Test sample**
23 February 2011	Eleutherococcus extract siccum

Customer's journal no/ project title:

-

Label(s): Batch no. 1521077

Information from client: -

Methods: Specific references are listed with the test results.

Reference: -

Results: Results are reported on page 2-3.

Comments: Samples are analysed by the laboratory:

Dr. Fintelmann und Dr. Meyer, GMBH, Germany

Signatures

Sandy Leth
MSc (Pharm)

eurofins | Fintelmann und Meyer GMP GmbH

Eurofins Fintelmann und Meyer GMP GmbH
Großmoorbogen 25
D-21079 Hamburg
GERMANY

Tel: +49 40 49 294 670
Fax: +49 40 49 294 699

info@fintelmann-meyer.de
www.fintelmann-meyer.de

Eurofins Fintelmann und Meyer GMP GmbH · Großmoorbogen 25 · D-21079 Hamburg

Svenska Ortimedicinska Institutet
attn. Mrs. Eva Vencel
Kövlingevägen 21
31250
SCHWEDEN

Report date 28.02.2011

Analytical report AR-11-FG-001467-01

Sample Code 714-2011-00002782

**Reference**	Eleutherococcus extrakt siccum Batch nr:15 21077 16.02.2011
**Sample sender**	Mrs. Eva Vencel
**Prescriber**	Eurofins Pharma A/S
**Reception date time**	24.02.2011
**Client sample code**	Our reference number:9408-4801
**Packaging**	plastic container
**Reception temperature**	room temperature
**Storage conditions**	room temperature
**Start analysis**	24.02.2011
**End analysis**	28.02.2011

Test results

FGL15 Ethanol
Method EP 7.0, on the basis of method 2.4.24

Ethanol	<1	%

JUDGEMENT

With regard to the analysed parameters the tested sample material meets the respective requirements of the customer.

"Analysis required by Eurofins Pharma A/S"

Fintelmann und Meyer GMP GmbH

Analytical report AR-11-FG-001467-01
Sample Code 714-2011-00002782

Signature

Dr. H. Neumann-Hensel / B. Kohnke / K. Oelkers / Dr. T. Rathjen
(General manager) (Department management)

eurofins
Eurofins Pharma A/S
Strandesplanaden 110
2665 Vallensbæk Strand
T. 70 22 42 96

2011.04.07

01. Mar. 2011

eurofins

Fintelmann und Meyer GMP GmbH

Eurofins Fintelmann und Meyer GMP GmbH
Großmoorbogen 25
D-21079 Hamburg
GERMANY

Tel: +49 40 49 294 670
Fax: +49 40 49 294 699

info@fintelmann-meyer.de
www.fintelmann-meyer.de

Eurofins Fintelmann und Meyer GMP GmbH · Großmoorbogen 25 · D-21079 Hamburg

Svenska Ortimedicinska Institutet
attn. Mrs. Eva Vencel
Kövlingevägen 21
31250
SCHWEDEN

Report date 28.02.2011
Page 1/2

Analytical report AR-11-FG-001467-01

Sample Code 714-2011-00002782

**Reference**	Eleutherococcus extrakt siccum Batch nr:15 21077 16.02.2011
**Sample sender**	Mrs. Eva Vencel
**Prescriber**	Eurofins Pharma A/S
**Reception date time**	24.02.2011
**Client sample code**	Our reference number:9408-4801
**Packaging**	plastic container
**Reception temperature**	room temperature
**Storage conditions**	room temperature
**Start analysis**	24.02.2011
**End analysis**	28.02.2011

Test results

FGL15 Ethanol
Method EP 7.0, on the basis of method 2.4.24

Ethanol	<1	%

JUDGEMENT

With regard to the analysed parameters the tested sample material meets the respective requirements of the customer.

"Analysis required by Eurofins Pharma A/S"

2011.03.01
[illegible]

Fintelmann und Meyer GMP GmbH

Analytical report AR-11-FG-001467-01
Sample Code 714-2011-00002782

Signature ______________________________ i.V. [illegible] i.V. [illegible]

Dr. H. Neumann-Hensel / B. Kohnke / K. Oelkers / Dr. T. Rathjen
(General manager) (Department management)

eurofins
Eurofins Pharma A/S
Strandesplanaden 110
2665 Vallensbæk Strand
Tlf. 70 22 42 96

01. Mar. 2011

Svenska Örtmedicinska Institutet
Eva Vencel
Kövlingevägen 21
SE-312 50 Vallberga

Eurofins Pharma A/S
Strandesplanaden 110
DK-2665 Vallensbæk Strand
Denmark

Tel. **+45 70 22 42 66**
Fax **+45 70 22 42 55**
eurofins@eurofins.dk
www.eurofins.dk

Date
01 March 2011
Our ref.
940848/SLH

Analytical report

**Sample received**	**Test sample**
23 February 2011	Eleutherococcus extract siccum

Customer's journal no/ project title:

-

Label(s): Batch no. 1521077

Information from client: -

Methods: Specific references are listed with the test results.

Reference: -

Results: Results are reported on page 2-3.

Comments: Samples are analysed by the laboratory:
Dr. Fintelmann und Dr. Meyer, GMBH, Germany

Signatures

Sandy Leth
MSc (Pharm)
