# Supplementary material for: Early intervention with Kan Jang® to treat upper-respiratory tract infections: A randomized, quadruple-blind study
Source: J Tradit Complement Med. 2021 Jun 11;11(6):552–62. doi: 10.1016/j.jtcme.2021.06.001 (PMC8572720; doi:10.1016/j.jtcme.2021.06.001)
Supplement: Multimedia component 4 [file mmc4.pdf]

SAFETY EVALUATION

Extent of Exposure

Duration: 1-5 days

Dose: 6 capsules daily, in total of 935 mg of fixed combination of 866.4 mg proprietary *Andrographis paniculata* L. Nees. herb standardized native extract , and 68.4mg proprietary *Eleutherococcus senticosus* root native extract, equivalent to 5.8 mg of Herba Andrographidis, and 1.2 g of Radix Eleutherococci.**2**

Adverse Events (AEs)

Summary of adverse events

The treatment was well tolerated. The only common side effect was a mild pruritus (itchy skin), observed in 10 patients: 6 in the placebo group, and 4 - in the Kan Jang group. Serious adverse events were not observed.

Display of adverse events

Adverse events occurring after initiation of study treatments, including events likely to be related to the underlying disease or likely to represent concomitant illness are displayed in summary tables 1 and 2.

No adverse events of allergic reactions like urticaria, angioedema, paresthesia, anaphylactic reactions, rush were observed.

Analysis of adverse events

Analysis of adverse events observed in placebo and verum groups (Tables 1 and 22) shows that:

- the types adverse events are the same o in both groups
- the frequency (in %) is the same o in both groups.

In can be concluded that these events are not related to the treatment..

Deaths, Other Serious Adverse Events, and Other Significant Adverse Events

Deaths, other serious adverse events, and other significant adverse deserve special attention have not been observed in this study.

Clinical Laboratory Evaluation

The results of blood analysis, table 19, shows normalizing (anti-inflammatory) effect of Kan Jang.

Vital Signs, Physical Findings, and Other Observations Related to Safety

Vital signs related to safety do not reveal any evidence of a drug effect.

Safety Conclusions

The treatment was well tolerated. The only common side effect was a mild pruritus, observed in 10 patients: 6 in the placebo group, and 4 - in the Kan Jang group. Serious adverse events were not observed.

Table 1. Adverse events: treatment emergent signs and symptoms" (TESS) - those not seen at baseline. Number observed and rate with patient identification. Kan Jang group, N=94

Adverse event	Mild		Moderate		Severe		Total		Total
	Possibly Related	NR*	Possibly Related	NR	Possibly Related	NR	Possibly Related	NR	R+NR *
Pruritus/ itchy skin		4(4.2%)<br>N47**<br>N51<br>N46<br>N160							4 (4.2%)

** NR = not related; R – possibly related ** Patient identification number*

Table 2. Adverse events: treatment emergent signs and symptoms" (TESS) - those not seen at baseline. Number observed and rate with patient identification. Placebo group, N=85

Adverse event	Mild		Moderate		Severe		Total		Total
	Possibly Related	NR*	Possibly Related	NR	Possibly Related	NR	Possibly Related	NR	R+NR
Pruritus/ itchy skin		6 (7.0%)<br>N7**<br>N77<br>N66<br>N196<br>N36<br>N10							6 (7.0%)

Table 3. Overall summary of the treatment-emergent adverse events

	Group B (Kan Jang)	Group A (Placebo)	Total	Odds ratio, B/A	z statistic	Significance level, *p* value
Number of patients	94	85	179			
Patients with AE	4 (4.2%)	6 (7.1%)	10 (5.6%)	**0.5852**	**0.807**	**0.4194**
Total number of AEs: pruritus/ itchy skin	4 (4.2%)	6 (7.1%)	10 (5.6%)			
Number of patients with other serious AEs	0 (0%)	0 (0%)	0 (0%)			
Number of patients withdrawn for AEs	0 (0%)	0 (0%)	0 (0%)			
Cases of death due to AEs	0 (0%)	0 (0%)	0 (0%)			
