# Supplementary material for: Early intervention with Kan Jang® to treat upper-respiratory tract infections: A randomized, quadruple-blind study
Source: J Tradit Complement Med. 2021 Jun 11;11(6):552–62. doi: 10.1016/j.jtcme.2021.06.001 (PMC8572720; doi:10.1016/j.jtcme.2021.06.001)
Supplement: Multimedia component 5 [file mmc5.pdf]

SUPPLEMENT 4

Table 1. Baseline blood hematology analysis results at the beginning of URI , patients included in the study

	**Placebo, n=85**		**Kan Jang, n=94**		**p-value**
	Mean	SE	Mean	SE	
Hb g/l	**140.5**	13.950	**125.0**	1.409	0.5148
RBC 1012/l	**9.532**	1.360	**11.42**	1.477	0.5905
FI	**0.933**	0.0053	**0.931**	0.0048	0.8812
WBC 109/l	**9.649**	0.1023	**9.441**	0.1211	0.4629
Stab neutrophils %	**4.071**	0.1541	**4.277**	0.143	0.3284
Segmented neutrophils %	**60.72**	0.9282	**62.09**	0.8236	0.3284
Eosinophils %	**3.729**	0.09815	**3.851**	0.0855	0.3704
Lymphocytes %	**18.95**	0.2845	**19.14**	0.3102	0.6823
Monocytes %	**3.259**	0.0583	**3.333**	0.0558	0.5647
ESR mm/h	**22.73**	0.4366	**23.36**	0.3488	0.4638

WBC – white blood cells, STN and SGN - stab and segmented neutrophils, EOS – eosinophils, L- lymphocytes, M- monocytes, ESR – erythrocytes sedimentation rate, FI- farb index.

Table 2. Changes from the baseline of blood hematology analysis results at the endpoint day 5

	**Placebo, n=85**		**Kan Jang, n=94**		**p-value**
	Mean	SE	Mean	SE	
FI	-1.753e-01	0.003	-0.002	0.005	0.992
WBC 10^9/l	-0.549	0.108	-0.706	0.120	**0.007****
Stab neutrophils %	-0.659	0.113	-0.723	0.112	0.604
Segmented neutrophils %	-0.235	-0.235	0.681	0.591	0.637
Eosinophils %	-0.435	0.068	-0.425	0.063	0.782
Lymphocytes %	-0.047	0.202	0.702	0.261	0.149
Monocytes %	-0.212	0.053	-0.298	0.052	0.407
ESR mm/h	-2.118	0.223	-2.819	0.208	**0.026***

WBC – white blood cells, STN and SGN - stab and segmented neutrophils, EOS – eosinophils, L- lymphocytes, M- monocytes, ESR – erythrocytes sedimentation rate, FI- farb index
