# Supplementary material for: Early intervention with Kan Jang® to treat upper-respiratory tract infections: A randomized, quadruple-blind study
Source: J Tradit Complement Med. 2021 Jun 11;11(6):552–62. doi: 10.1016/j.jtcme.2021.06.001 (PMC8572720; doi:10.1016/j.jtcme.2021.06.001)
Supplement: Multimedia component 6 [file mmc6.pdf]

SUPPLEMENT 6

Figure 1. Inhibitory effects of Andrographis, Eleutherococcus extracts and their combination (Kan Jang) on molecular network associated with anti-inflammatory and antimicrobial response. Andrographys downregulated 11 of 28 genes lead to predicted inhibition of inflammation, only 4 genes are downregulated by Eleutherococcus, while in combination (Kan Jang) they synergistically deregulate 28 of 28 genes of inflammatory network.

* - For references see Panossian et al. 2015; https://doi.org/10.1016/j.phymed.2015.08.004

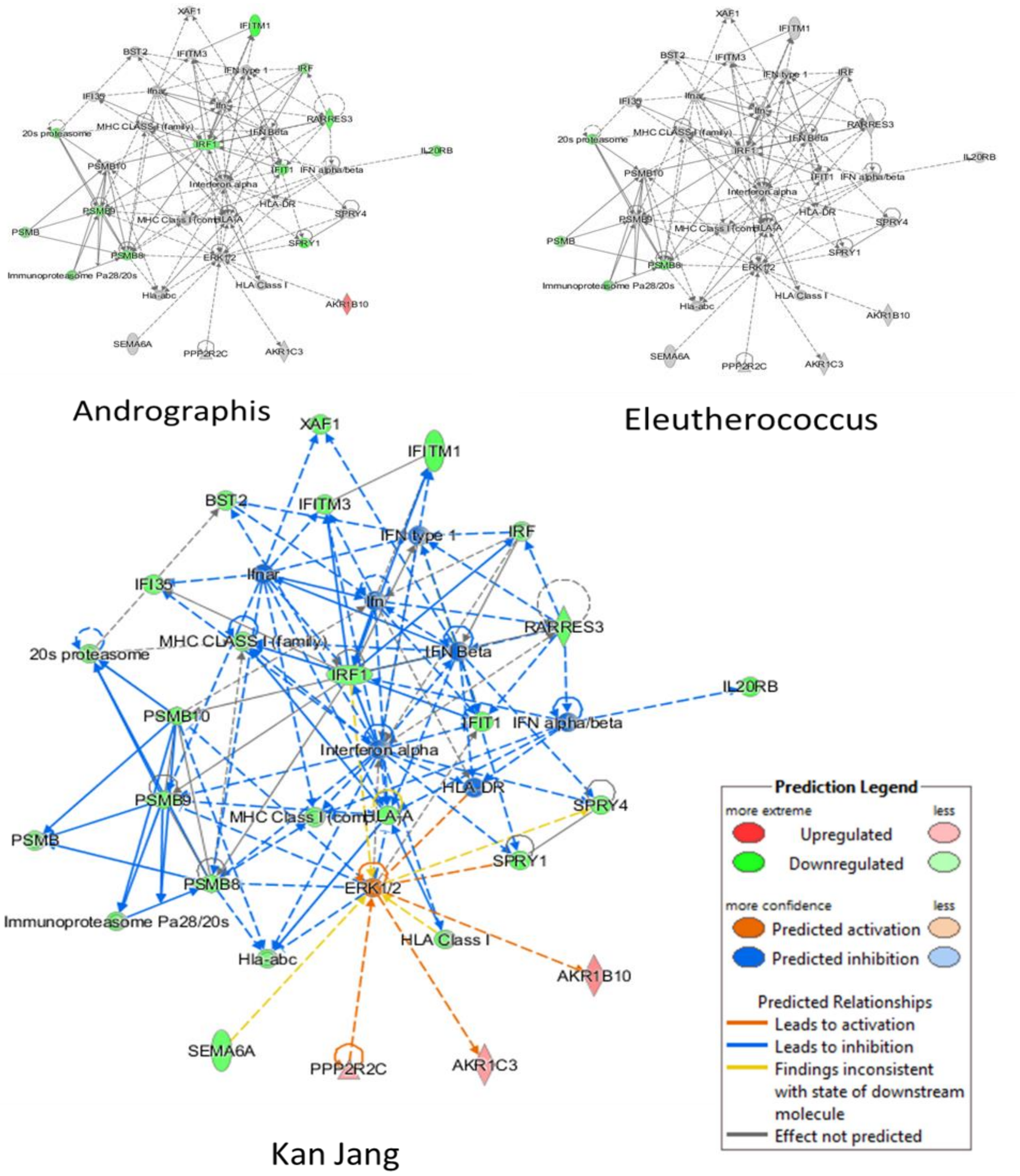

A summary of findings from pre-clinical and studies on the mechanism of action of Andrographis and Eleutherococcus that potentially have clinical significance in relieving the symptoms of upper respiratory tract infections.

Andrographis paniculata extracts and their active constituents have polyvalent actions targeting multiple mediators involved in the life cycle of viruses and bacteria and acute inflammatory host response to an infectious agent.

Polyvalent action of Andrographis includes:

- anti-inflammatory activity associated with modulation of immune response of the innate immune system by inhibition of NFk-B, platelet-activating factor (PAF) antagonism, inhibition of NO production in macrophages, inhibition of histamine and proinflammatory interleukins,
- antiviral activity (associated with the direct inhibition of replication of viruses and indirect antiviral effect via activation of the immune system, including cytokines formation)
- antibacterial activity (associated with the direct bacteriostatic and bactericide effects and indirect antimicrobial effect via activation of the innate and adaptive immune response).

The pharmacology of *A. paniculata* extracts and their active principles, primarily andrographolides I-III, has been studied in different animal models using different modes of administration (oral, intravenous, subcutaneous) and different species including mouse, rat, pigs, rabbit, dog, monkey and isolated tissues from different species, bacteria *in vitro.*

These studies have demonstrated an effect on:

- **an immune system** - anti-inflammatory (including anti-pyretic, analgesic and anti-allergic) effect, IFɣ - mediated antibacterial and antiviral effects, NF-κB, TNF-α, IL-1, IL-2, IL-6, IL-12, COX-2, PAF, LTB_4 and NO mediated effects on the respiratory system,
- **a neuroendocrine system** – analgesic effect,
- **an infectious agent** - antiviral and antimicrobic effects.

Table 1. Viricidal effects

Virus name	Eleutherococcus<br>eleutherosides	Andrographis<br>andrographolies
SARS-COV-2		Asea et al., 2021
human rhinovirus (HRV),	Glatthaar-Saalmuller et al., 2001	
respiratory syncytial virus (RSV)	Glatthaar-Saalmuller et al., 2001	
H1N1 influenza A virus	Glatthaar-Saalmuller et al., 2001<br>Yan et al. 2018,<br>Yan et al. 2020,	Ding et al. 2017<br>Yu et al., 2014<br>Ko, Wei and Chiou 2006
H5N1 avian influenza virus		Sornpet et al, 2917
Chikungunya virus		Wintachai et al., 2015
Dengue virus		Panraksa et al., 2017;<br>Ramalingam et al. 2018;

* - For references see Panossian and Brendler 2020;. https://doi.org/10.3390/ph13090236

Table 2. Specific Antiviral Action: Effects on SARS virus binding to cells and replication

Target/mediator	Eleutherococcus<br>eleutherosides	Andrographis<br>andrographolides
Effects on viral life cycle in infected host cells – targets preventing the virus RNA synthesis and replication		
Nsp5: 3-chymotrypsin-like protease (3Clpro) – M^{pro} main protease of SARS-COV-2	Zhang et al., 2020<br>Mani et al. 2020	Enmozhi et al., 2020;<br>**Wu et al., 2020**
Nsp3: papain like protease (Plpro)	Zhang et al., 2020<br>Mani et al. 2020	**Wu et al., 2020**
Nsp12: RNA-dependent RNA polymerase (RdRp)		**Wu et al., 2020**
Nsp1: the most N-terminal gene 1 protein		Wu et al., 2020
Targets inhibiting virus structural proteins		
S2: Spike glycoprotein receptor to type-II transmembrane serine protease enzymes (TMPRSS2) of host cells		Wu et al., 2020

* - For references see Panossian and Brendler 2020;. https://doi.org/10.3390/ph13090236

Table 3. Non-Specific Antiviral Action: Effects on innate and adaptive immunity

Target/mediator	Eleutherococcus<br>eleutherosides	Andrographis<br>andrographolides
Innate Immunity		
Defensins peptides<br>Human β-defensin-2		Shao, et al. 2012;<br>Xiong et al. 2015
Pathogen's pattern recognition receptors TLR proteins	Panossian et al.,2018;<br>Han et al., 2003;	Gao and Wang, 2016;<br>Kim et al., 2018
Interferons		Panossian et al., 2002
Interleukins: IL-6, IL-1β, IL-10, TNF etc.	Jin et al. 2020;<br>Panossian et al., 2003	Chao, et al. 2011<br>Panossian et al., 2003;<br>Panossian et al., 2002
Melatonin signaling pathways	Panossian et al.,2018;	
Adaptive immunity		
B Cells and Antibodies	Han et al., 2003;	Panossian et al., 2002

* - For references see Panossian and Brendler 2020;. https://doi.org/10.3390/ph13090236

Table 4. Anti-inflammatory effect, reparations of oxidative stress induced injuries in compromised cells and tissues

**Target/mediator**	**Eleutherococcus** eleutherosides	**Andrographis** andrographolides
Arachidonic acid release, inhibition of phospholipase 2		Kishore et al., 2016
COX-2 mediated signaling	Panossian et al.,2019	Chao, et al. 2009; Kim et al., 2018
Lipoxygenases mediated signaling of arachidonic acid pro- and anti-inflammatory metabolites leukotrienes, lipoxines, resolvins, etc.	Panossian et al.,2019	
PAF: platelet activating factor		Amroyan et al., 1999; Burgos et al., 2005;
Nitric oxide mediated inflammation: inducible NO synthase oxide catabolites (NOC)	Panossian et al., 2003; Panossian et al., 2007	Chiou, Chen and Lin 2000 Panossian et al., 2003
NFkB mediated inflammation NFkB signaling, translocation and expression	Han et al., 2003;	Dai et al. 2019; Chao, et al. 2011; Gao and Wang, 2016; Kim et al., 2018
Nrf2-mediated Oxidative Stress Response Signaling Pathway proteins: Phosphatidylinositol 3-kinase (PI3K), protein kinase B (Akt), KEAP1, etc. Nrf2-ARE (antioxidant response element) -expression	Wang et al., 2010	
Antioxidant proteins (SOD, GST, NQO1 and HO1), lipids peroxidation	Wang et al., 2010;	
Molecular chaperons mediated cytoprotecting, and repairing processes Heat shock proteins Hsp72,	Panossian et al., 2007 Asea et al., 2013; Panossian et al., 2009	
Melatonin signaling Retinoic-acid-receptor (RAR)-related orphan nuclear receptor alpha (RORα),	Panossian et al.,2018	

* - For references see Panossian and Brendler 2020;. https://doi.org/10.3390/ph13090236

A summary of findings from pre-clinical and studies

Systematic reviews of available clinical studies suggest that *A. paniculata* is significantly superior to placebo in relieving the symptoms of uncomplicated URTI and in shortening the time to symptom resolution.[19-27] Prophylactic treatment with the *E. senticosus* extract has also been shown to reduce a number of complications associated with the influenza infection, including pneumonia, bronchitis, and otitis, as well as morbidity and mortality rates.[5,6]

Table 5. Clinical efficacy in respiratory tract infectious diseases

Eleutherococcus	Andrographis
EMA/HMPC, 2013;	Hancke et al. 1995;
Galanova, 1977;	Caceres et al. 1999;
Schezin et al., 1977;	Melchior et al. 1996;
Gagarin IA, 1977;	Saxena et al. 2010
Barkan et al.,1980;	Panossian and Wikman, 2012
Shadrin et al., 1986;	
Sheparev et al., 1986	
**Kan Jang combination**	
Caceres et al. 1997; Gabrielyan et al., 2000; Melchior et al., 2000; Kulichenko et al., 2003; Spasov et al., 2004,	

* - For references see Panossian and Brendler 2020;. https://doi.org/10.3390/ph13090236
